# NanoTraPPED—A New Method for Determining the Surface Energy of Nanoparticles via Pickering Emulsion Polymerization

**DOI:** 10.3390/nano11123200

**Published:** 2021-11-25

**Authors:** Andrei Honciuc, Oana-Iuliana Negru

**Affiliations:** Electroactive Polymers and Plasmochemistry Laboratory, “Petru Poni” Institute of Macromolecular Chemistry, Aleea Gr. Ghica Voda 41A, 700487 Iasi, Romania; negru.oana@icmpp.ro

**Keywords:** Pickering emulsions, Pickering emulsion polymerization, functionalized nanoparticles, surface energy, polar and dispersive surface energy, interfacial adsorption of nanoparticles

## Abstract

Surface energy with its polar and disperse components describes the physicochemical state of nanoparticles’ (NPs) surfaces, and can be a valuable parameter for predicting their bulk behavior in powders. Here, we introduce a new method, namely, Nanoparticles Trapped on Polymerized Pickering Emulsion Droplets (NanoTraPPED), for measuring the surface energy of a series of silica NPs bearing various surface functional groups. The method consists in creating Pickering emulsions from vinyl bearing monomers, immiscible with water, whereas NPs of interest have a stabilizing role, and in the process, become trapped at the monomer/water interface of emulsion droplets. The Pickering emulsion is polymerized, and polymer microspheres (colloidosomes) decorated with NPs are obtained. NanoTraPPED relies on measuring contact angles from the immersion depth of nanoparticles at the interface of various polymer colloidosomes with the electron microscope. The contact angle values are used as input for the Owens-Wendt-Rabel-Kaelble (OWRK) model, to quantitatively determine the total surface energy with water γ_NP/water_, air γ_NP_, and the corresponding polar and dispersive interaction components of NPs carrying -NH_2_, -SH, -OH, -CN and -C8 surface functional groups, ranking these according to their polarity. Our findings were confirmed independently by calculating the interfacial desorption energies of NPs from contact angles.

## 1. Introduction

Nanoparticles’ (NPs) surface functional groups that control their surface reactivity may also, at least in part control their behavior, such as their ability to aggregate, disperse, attach on surfaces, flow, etc. The surface energy of the NPs, which can be broken down into the contributions of various types of components—polar, dispersive, hydrogen bonding, acidic, basic, etc. [1,2,3,4,5]—can serve as one of the essential parameters to describe the surface physicochemical state of the NPs, predict their behavior in bulk, and ultimately enable a holistic classification of NPs into various categories. While, establishing causal relationships between various components of the surface energy and the bulk behavior of NPs in powders represents an ongoing challenge for fundamental science, it also has great technological importance for the design of drug formulations with enhanced bioavailability [6,7], flowing ability [8], dispersing of pigments in paints or fillers in polymer matrices [9], heterogeneous catalysis [10,11] and reactor design [12], stabilization of reactive nanopowders [13], powder cohesion [14], flotation of ore minerals [15,16,17], emulsification [18], gauging the amphiphilicity of colloids [19], etc.

Surface energy is trivial to measure on macroscopic surfaces and is a parameter monitored in many industries: when determining the adhesion of a photoresist on a silicon wafer in integrated circuit manufacturing [20,21], coating the metal parts [22] in the auto industry, and designing functional surfaces in nanotechnology [23,24,25].

For NPs, however, the surface energy and its components are extremely challenging to determine unambiguously with the standard methods. The surface energy models [1,2,3,4,5] first require measuring the contact angle with several liquids; this could be done with a sessile liquid droplet [19] on pelleted powders and films or with the more established capillary rise methods, such as the Washburn method [26], or using the thin layer wicking technique [27,28]. These bulk methods sometimes fail due to technical challenges. Thus, significant effort was invested into the development of new single-particle methods for determining the contact angles, but they all require more advanced equipment and suffer from some limitations. For example, direct measurement of the contact angle of single particles via colloidal probe-atomic force microscopy (AFM) [29] is rather limited to micron-sized particles; the gel trapping techniques (GTT) [30] relies on NPs’ spontaneous interfacial adsorption and is limited to NPs larger than the length scale of gelation; and freeze-fracture shadow casting (FreSCA) [31] relies on NPs with low activation energy to interfacial adsorption, and requires water vitrification.

We have developed a new method, namely, Nanoparticles Trapped on Polymerized Pickering Emulsion Droplets (NanoTraPPED), for determining the surface energy of nanoparticles by employing a new strategy to measure the contact angle, based on trapping NPs at the interface between a polymer and water via Pickering emulsion polymerization. This method involves first the creation of oil-in-water (o/w) Pickering emulsions; the oil droplets consist of a monomer, typically vinyl bearing monomers, immiscible with water. The Pickering emulsion is subsequently polymerized, and the resulting solid polymerized droplets, i.e., colloidosomes, have nanoparticles (NPs) trapped on their surfaces. The surface trapped NPs can be mechanically or chemically removed. From the traces left on the surfaces of the colloidosomes, one can measure the immersion depth of an NP and thus determine the contact angles of the NP with the polymer. By measuring the contact angle of the same NP on several different polymers, varying in polarity, the total surface energy, and its components—the dispersive, polar, hydrogen, and acidic or basic—can be calculated. The relative magnitudes of the surface energy components reflect the preferred modes of interaction of the surface of the nanoparticle with other NPs or with the environment [4]. Thus, the physicochemical state of the surface of the particle can be determined and characterized according to relative ratios of the surface energy components. This new NanoTraPPED technology could be universally applicable to all types of nanoparticles, from polymeric and metallic to ceramic and composites which are capable of stabilizing Pickering emulsions, meaning they must adhere to the interface.

## 2. Materials and Methods

### 2.1. Materials

Tetraethylorthosilicate (TEOS) 99%, trimethoxy(octyl)silane 97% (OTS), 3-(mercaptopropyl)trimethoxy silane (MPTMS), and benzoin methyl ether (BME) 97% were purchased from abcr GmbH, Karlsruhe, Germany. 3-(Triethoxysilyl) propionitrile 97% (TESPN), 3-(trimethoxysilyl) propylamine 98% (APTES), styrene (Sty) ≥ 99% containing 4-tert-butylcatechol as a stabilizer, divinylbenzene (DVB) technical grade—80%, benzyl metacrylate (BM) 96% containing monomethyl ether hydroquinone as an inhibitor, tert-butyl acrylate (tBA) 98% containing 10–20 ppm monomethyl ether hydroquinone as an inhibitor, vinylacetate (VAc) 99% containing 3–20 ppm hydroquinone as an inhibitor, methyl methacrylate (MM) 99% stabilized for synthesis with monomethyl ether hydroquinone as an inhibitor, 2-(dimethylamino)ethyl methacrylate (DAEMA) containing 700–1000 ppm monomethyl ether hydroquinone as an inhibitor—98%, ethyl methacrylate (EM) 98% stabilized with 200 ppm hydroquinone, dimethylsulfoxide (DMSO) anhydrous 99.9%, diiodomethane Reagent Plus 99% containing copper as stabilizer, and aluminum oxide (active basic) Brockmann I were purchased from Sigma-Aldrich. Ethanol absolute (EtOH, 99.3%) and hydrochloric acid (HCl) were purchased from Chemical Company S.A. Iasi, Romania; ammonium hydroxide solution (28–30%) for analysis EMSURE ACS. Reag. Ph Eur. Supelco were purchased from Sigma-Aldrich (Merck KGaA, Darmstadt, Germany); ethylene glycol ≥ 99% from AlfaAesar (ThermoFischer GmbH, Kandel, Germany); formamide 99.5% from Acros Organics, Geel, Belgium.

### 2.2. Synthesis and Characterization of Silica Nanoparticles

In a 1000 mL round-bottom flask, 6 mL TEOS, 300 mL EtOH, 22 mL H_2_O, and 18.5 mL NH_4_OH were added. This reaction mixture was stirred at room temperature at 1000 rpm. A second mixture of 36 mL TEOS and 150 mL EtOH was added drop-wise via a separatory funnel over about 2 h. The reaction mixture was left for 20 h at room temperature. At the end, the reaction mixture was neutralized with 12 mL of HCl. The silica nanoparticles were washed by dispersion in fresh solution, which was followed by centrifugation. The particles were washed in this way three times with EtOH and three times with water. The separation was done by centrifugation for 4 min, at 5500 rpm, in a Hermle Z 326K centrifuge (HERMLE Labortechnik GmbH, Wehingen, Germany) at 0 °C. At the end, the particles were dispersed in EtOH. The nanoparticle diameter was determined with a Verios G4 UC scanning electron microscope (SEM) (Thermo Fischer Scientific Inc., Eindhoven, The Netherlands) equipped with an energy dispersive spectrometer (EDS), EDAX Octane Elite (EDAX LLC/AMETEK, Mahwah, NJ, USA); see Figure S1 in the Supplementary Material. The zeta potential ζ of the pristine silica nanoparticles was determined with a Zetasizer NanoZS (Malvern Panalytical Ltd, Malvern, UK) to be ζ = −49.7 ± 1.4 mV, and the diameter D = 500 ± 7 nm by SEM; see Table S1 in the Supplementary Material.

### 2.3. Surface Functionalization of Silica Nanoparticles

First, 1.2 g of silica nanoparticles (20 mL of EtOH) was poured in a 100 mL flask. Then, 30 mL of EtOH was added and the mixture was kept under a nitrogen atmosphere. The reaction was stirred at 1000 rpm. Next, 2 mL of silane coupling agents was added drop by drop; the chemical structures of the surface modifying agents are shown in Figure S2. At the end of the addition time, the reaction mixture was heated and maintained to 60 °C for 20 h. The functionalized nanoparticles were washed three times with EtOH, three times with H_2_O, two times with EtOH, and another two times with H_2_O, and were finally redispersed in H_2_O. The samples were analyzed with SEM (Figure S3 in the Supplementary Material), which showed no morphological changes after functionalization. The zeta potentials before and after modification are given in Table S1. The FTIR showing successful functionalization of silica nanoparticles is shown in Figure S4 in the Supplementary Material. The energy dispersive X-ray (EDX) analysis presented in Figure S5 proves the successful functionalization of the NP-SH, NP-CN. Further, the ninhydrine test in Figure S6 proves the presence of the amine functional group in NP-NH_2_.

### 2.4. Pickering Emulsion Preparation and Polymerization

Water immiscible vinyl bearing monomers MM, EM, BM, DAEMA, tBA, VAc, and Sty, having different polarities (chemical structures are given in Figure S7 in the Supplementary Material), and a crosslinking monomer DVB, were used for the Pickering emulsion preparation and polymerization. For the preparation of Pickering emulsions, 20 mg of a BME radical initiator was weighed in a 20 mL glass scintillator vial; then 1 mL monomer was added, followed by 0.1 mL DVB crosslinker; the mixture was left 5 min to produce a homogeneous solution; finally, 5 mg colloidal particles and 12 mL water were added. Next, the glass scintillator vial was sonicated for 15 s at 30% amplitude with a Branson 450 Sonifier (BRANSON Ultrasonics Corporation, Danbury, Connecticut, USA) equipped with a 7 mm diameter horn. Subsequently, the prepared Pickering emulsions were placed under the UV lamp (wavelength = 365 nm, with 4 lamps, each with an intensity = 2.2 mW/cm^2^) for 1 h to initiate the polymerization reaction. The detailed recipe corresponding to each monomer is given in Table S2 in the Supplementary Material. At the end of the reaction time, the polymerization product was filtered and washed with 10 mL EtOH to remove the unreacted monomer, and left to dry at room temperature. Typical features observed after the polymerization reaction were colloidosomes, which resulted from the oil-in-water (o/w) Pickering emulsions. The following polymer colloidosomes were obtained: polystyrene (PSty), poly (methyl methacrylate) (PMM), poly (ethyl methacrylate)(PEM), poly (tert-butyl methacrylate)(PtBA), and poly (benzyl methacrylate)(PBM), poly(vinyl acetate) (PVAc) and poly(2-(dimethylamino)ethyl methacrylate)( PDAEMA). They were microspheres decorated each with a layer of stabilizing nanoparticles. The polymerized Pickering emulsion could be observed with a Leica DM 2500 M (Leica Microsystems GmbH, Wetzlar, Germany) inverted optical microscope; see Figures S8–S12 for NP-C8, NP-CN, NP-NH2, NP-SH, and NP-OH. To remove the nanoparticles trapped at the interface, the samples were then sonicated in an ultrasonic bath for 1 min, which was sufficient to remove the nanoparticles that had shallow immersion depths in the polymer. However, in order to remove the nanoparticles with higher interfacial immersion depth into the polymer and because ultrasonication was not powerful enough, the silica nanoparticles were successfully removed by leaving the samples for 24 h in an aqueous solution of NaOH at pH 13, as shown in the SEM images, before and after treatment of the colloidosomes. This way, most of the nanoparticles trapped on the polymer’s surface could be successfully removed. Further, the colloidosomes obtained after the Pickering emulsion polymerization were analyzed with SEM after sputtering of 4 Å Pt.

### 2.5. Measurement of the Contact Angle

The interfacial immersion depth of the nanoparticles trapped at the polymer-water interface during the Pickering emulsion formation and polymerization was studied with a Verios G4 UC (Thermo Fischer Scientific Inc., Eindhoven, The Netherlands) scanning electron microscope in field immersion mode, using either a Through-Lens-Detector (TLD) or a Mirror Detector (MD), using a stage bias of 1000 mV, a beam energy between 500 and 1 KeV, and an aperture of 500 pA–1 nA, to minimize the surface charging effects and also the impact of the electron beam on the polymer. The contact angle was determined from the circular traces left on the polymer’s surface by the nanoparticles.

## 3. Results

A series of reference silica nanoparticles, NP-OH, were prepared, and subsequently, their surfaces were chemically modified with different surface agents.

### 3.1. Synthesis of Silica Nanoparticles

Silica nanoparticles were prepared by hydrolysis and polycondensation of TEOS in water and ethanol upon addition of ammonium hydroxide; see Scheme S1 in the Supplementary Material.

### 3.2. Functionalization of Silica Nanoparticles

**Scheme 1:**
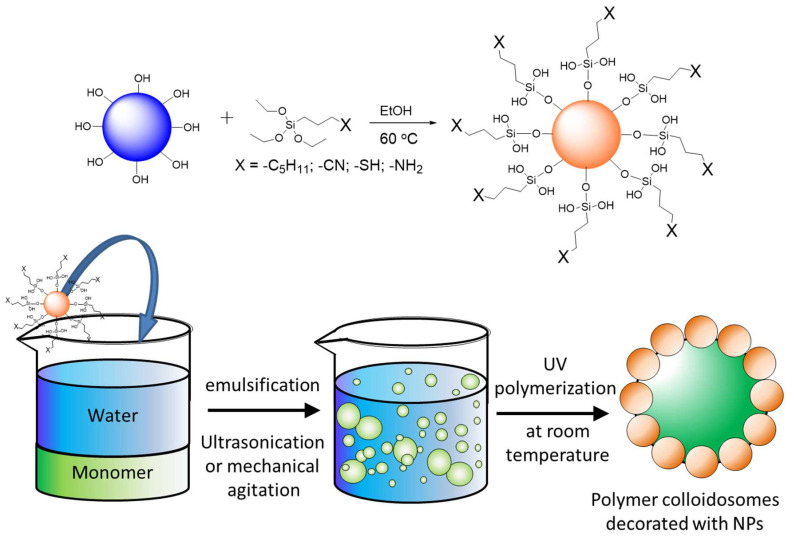
(**Top**) Reaction scheme for the surface functionalization, resulting in nanoparticles bearing different surface functional groups: NP-C8, NP-CN, NP-SH, NP-NH_2_. (**Bottom**) NPs were further employed in the stabilization of Pickering emulsion from a vinyl bearing monomer immiscible with water, followed by polymerization, resulting in the formation of polymer colloidosomes that had NPs trapped on their surfaces.

The same NP-OH batch was used for further surface functionalization, with trimethoxy-saline or triethoxy-silane derivatives (Figure S2), according to Scheme 1, such that the final NPs possessed outer shells with different chemical groups, which differ in their polarity: NP-C8, NP-CN, NP-NH_2_, NP-SH. The chemical functional groups can be ranked in polarity based on their dipole moment, according to Table S3 in the Supplementary Material. Further, the FTIR and the zeta potential (ζ) attest to the successful modification of the starting NP-OH; see Figure S4 and Table S1 in the Supplementary Material.

### 3.3. Generation of Pickering Emulsions Choice of Monomers

Pickering emulsions are emulsions stabilized by particles of different sizes and were named after S.U. Pickering (1907). As NPs undergo adsorption at the interface between oil and water during strong agitation, the NPs act as emulsion stabilizers by creating a protective layer around the dispersed liquid droplets, preventing their coalescence. Pickering emulsions have greater kinetic stability than the emulsions stabilized by molecular surfactants; this is because NPs, once adsorbed at the interface, become irreversibly trapped—e.g., desorption energies are in the order of tens of thousands of kT [4]. For the preparation of Pickering emulsions, NPs are first dispersed in water, oil is added, and then the mixture is agitated. Typically, there is an activation energy barrier to interfacial adsorption owing to the fact that the NP’s surface must be partially dehydrated and re-solvated by the second phase [32]. Therefore, external energy input is required in the form of ultrasonication or mechanical stirring to overcome this high activation energy barrier to NP adsorption.

The first step towards preparing the system to enable us to determine the surface energy and its components, was to prepare a Pickering emulsion for each type of NP according to the recipes given in Table S2 in the Supplementary Material. These emulsions were used as stabilizers with water immiscible monomers, which differ in their polarity, MM, EM, BM, DAEMA, tBA, VAc, and Sty, as shown in Scheme 1 and Figure S5. For the conditions used, i.e., water-to-oil ratio, most of the NPs and monomers formed oil-in-water (o/w) emulsions. A detailed investigation into other types of emulsion phases was not carried out. Important to note is that the unlike the other methods that work in quiescent conditions where NPs with low activation energy to interfacial adsorption can be measured, with our method, due to external mechanical energy input during emulsion creation, even NPs with high activation energy barriers to interfacial adsorption can be measured, which greatly expands the range of NPs that can be analyzed with NanoTraPPED.

### 3.4. Pickering Emulsion Polymerization

**Figure 1:**
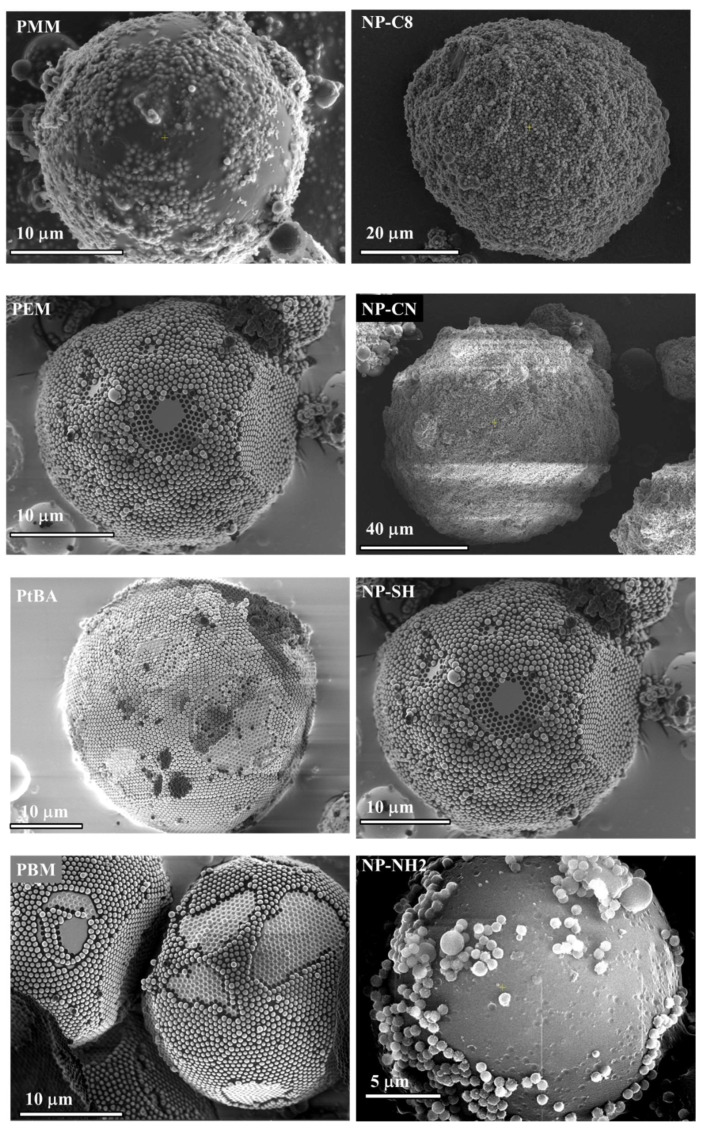
(**Left**) SEM images of different polymer colloidosomes, PMM, PEM, PtBA, and PBM, after the polymerization of Pickering emulsions stabilized by the same NP-SH. The colloidosomes clearly each have a layer of NPs at different interfacial immersion depths. (**Right**) SEM images of the same colloidosomes obtained from the same polymer (PEM) stabilized by different NPs: NP-C8, NP-CN, NP-SH, and NP-NH_2_, clearly showing different interfacial immersion depths.

The obtained Pickering emulsions were polymerized at room temperature under UV exposure according to the procedure described; see Scheme 1. After polymerization, the emulsion phase and successful polymerization were determined with an optical microscope. Typically, with the optical microscope, solid polymer microspheres or beads can be observed after the polymerization of o/w emulsions; see Figures S8–S12. Further investigation with the SEM revealed that the polymer microspheres were covered with a protective layer of self-assembled nanoparticles, which came from trapping of the emulsion stabilizing nanoparticles; see Figure 1 and Figures S8–S12.

**Figure 2:**
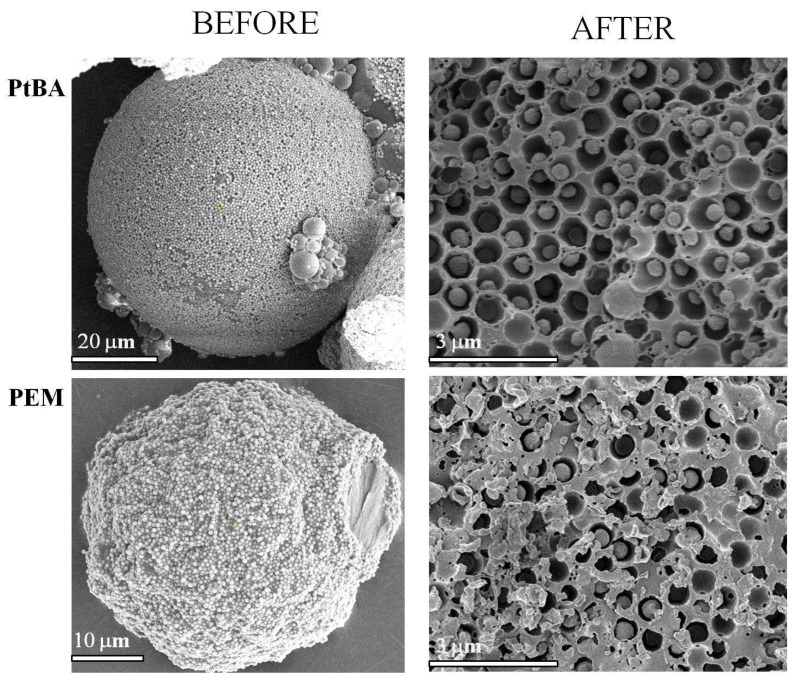
(**Left**): SEM images of the colloidosomes with the emulsion stabilizing nanoparticles trapped on their surfaces. (**Right**): SEM images of the surfaces of the colloidosomes after the removal of the nanoparticles with an aqueous NaOH solution at pH 13 for 24 h.

These structures are referred to as colloidosomes [33]. Some of the nanoparticles from the colloidosome’s surface can fall off after simple ultrasonication. If the immersion depth of the nanoparticles into the polymer surface was too deep, the silica nanoparticles could be removed by first treating the colloidosomes with a concentrated solution of NaOH for 24 h, which dissolved the NPs, leaving the circular traces perfectly observable with SEM; see Figure 2.

**Figure 3:**
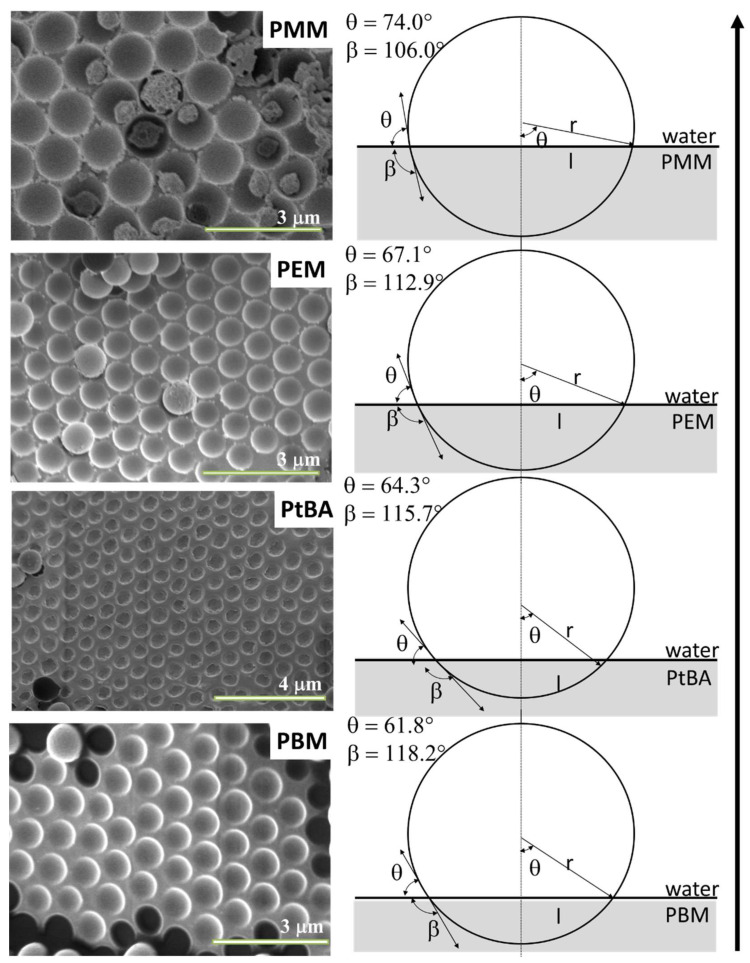
SEM images showing the circular traces left on various polymer colloidosomes, namely, PMM, PEM, PtBA, and PBM, by NP-SH, and the *θ* and *β* contact angles calculated using the following geometric relationship: $\theta =\sin^{-1}\frac{l}{r}$.

Traces of the nanoparticle could be seen on the colloidosome’s surface after nanoparticle removal; see Figure 3 (and Figures S8–S12). The radial size of the circular traces left on the colloidosome’s surface has direct relationships with the immersion depth and contact angle of the NPs on the polymer’s surface (see Figure 3); i.e., the larger the circular traces, the deeper the immersion depth of the nanoparticle into the polymer.

## 4. Discussion

The processes of Pickering emulsion formation and stabilization by NPs have been well described in previous works [4,34]. It is important to note that the phase of the obtained emulsion is determined by the NPs’ affinity to one phase or the other, according to Finkle [35] and Bancroft [36] rules. Thus, the surface energy of the NPs is the deciding factor [4]. For example, apolar low surface energy carbon black particles are more likely to form w/o emulsions than the high surface energy hydrophilic silica NPs, due to their greater affinity to the apolar phase than to water. Affinity of the NP to one phase or the other can be understood by the interfacial immersion depth of the NPs [4]; if the NP has a higher affinity for the oil phase than for water, then its immersion depth into oil at the oil-water interface will be greater than into water. In other words, the NP’s surface is wetted more strongly—i.e., the contact angle is lower—by one of the liquid phases than the other. Additionally, the interfacial immersion depth of the NPs will determine the curvature of the oil-water interface, and implicitly the emulsion phase. This is also the principle behind the design of pH-responsive Pickering emulsions previously reported by our group [32,37], where the surface energy modulation of the NPs due to the change in pH will change their interfacial immersion depth and lead to emulsion phase switching from w/o to o/w and vice versa. Thus, NanoTraPPED is a new method we have developed to visualize interfacial immersion depth of the NPs trapped at the interface, by polymerizing the emulsion droplets and thus creating a solid system, i.e., colloidosomes, that can be studied with electron microscopy techniques.

### 4.1. Measuring the Contact Angle of Nanoparticles at the Polymer-Water Interface

The contact angle of the nanoparticle with the polymer was determined from the from the SEM images from the radius of the void left on the colloidosome surface after nanoparticle removal; see the geometric relationship depicted in Figure 3. As the colloidosome structures are spherical, their curvature could make the measurements difficult; therefore, the SEM images were taken from a location typically from the apex of the colloidosome, as close as possible to an observation perspective perpendicular to the surface; see Figure 2 and Figure 3. There are two contact angles describing the interfacial immersion depth of the nanoparticle on the colloidosome’s surface, namely, *θ* and *β*; see Figure 3. For example, at the three-phase line, the two contact angles, *θ* measured from the water phase and *β* measured from the polymer phase, can be both calculated from the diameter of the circular trace observable on the colloidosome surface after nanoparticle removal. The principle of the NanoTraPPED method relies on the fact that the interfacial immersion depth, and consequently the magnitudes of the radii of the circular traces (contact angles *θ* and *β*) depend on the relative affinity, here defined by physical bonding strength, of the nanoparticle with each of the phases, water and polymer. Qualitatively, this trend can be seen from Figure 3, which shows that the NP-SH interfacial immersion depth into the polymer, and consequently the contact angle *θ*, increases as the affinity of the NP-SH for the polymer increases, from least polar PBM to most polar PMM; see Figure S13. Qualitatively, this indicates that NP-SH are rather polar and interact strongly with water, but as the polymer polarity increases, the NP-SH are drawn more and more toward the polymer phase; thus, *θ* increases and *β* decreases. The ranking of the polymer polarity is done by taking the ratio of the experimentally determined polar to disperse components, γPpγPd, (where $\gamma_{P}^{p}$ is the polar component of the surface energy of the polymer and $\gamma_{P}^{d}$ is the disperse component of the surface energy of the polymer), as shown in Table S4 and Figure S13; this type of ranking is valid, since the disperse component can never be zero. Thus, the polarity of the polymer increases in the following series: PtBA, PBM, PSty, PEM, PVAc, PMM, PDAEMA. The reason for the unusually high surface polarity of the PDAEMA could be the slight protonation in the acidic water pH = 5.6 after the radical polymerization.

Contact angle *β* is the contact angle of the NPs at the three-phase line of NP-polymer (NP/P), NP-water (NP/W), polymer-water (P/W), as indicated by the subscript of the Greek letter γ throughout this work. Why did not we consider the contact angle of the monomer instead? Initially, the oil droplets form a certain contact angle with the NPs. As soon as the polymerization starts, the contact angle will change until the equilibrium value with the polymer is reached, as the monomer is consumed, and more polymer is formed. The time scale needed for polymerization of the monomer droplets is 60 min, enough time for equilibration of the contact angle. In addition, the surface energy of the monomer and its homopolymer in the cases of those that are polymerized through radical polymerization are close. The ratio of the surface tension of the monomer to the surface energy of the polymer is proportional to the square of polymer density divided by the monomer density, typically a factor of 1.1 [38].

The values of the contact angles *β* of the functionalized NPs with the polymer, measured from the circular traces left on the colloidosome’s surface, are given in Table S5, from which some qualitative trends could be drawn. For example, the NP-C8 appears to have had the lowest contact angle *β* with the least polar polymer PtBA, implying a good affinity.

### 4.2. Adsorption Energy of the Nanoparticles

The adsorption energy and desorption energy can be calculated from the contact angles of the NPs at the three-phase line. For example, the energy required to remove a particle adsorbed at the PW interface into the water phase is given by [4,39,40]:

(1) $$\Delta E=\pi R^{2}\gamma_{PW}\left(1+\cos\beta \right)$$

While the above equation appears simple and convenient for determining the desorption energies of NPs, it is very difficult to apply because it relies on first measuring the contact angles of NPs at interfaces, which are difficult to measure other than via the Pickering emulsion polymerization, as was done here. In the past, to circumvent the lack of knowledge for the values of the contact angles of NPs at the interface, we used different methods to determine their interfacial desorption energies, but were strictly limited to NPs that could spontaneously adsorb at liquid/liquid or liquid/air interfaces [32,41]. In the current case, the clear advantage of NanoTraPPED comes from the fact that in a Pickering emulsion formation, the NP stabilizers that are more polar and typically have higher activation energy barriers to interfacial adsorption; the NPs are “forcibly” adsorbed at the interface by applying mechanical energy input. See the discussion in previous works [18,32,37,42]. Thus, the NanoTraPPED method applies to a wide polarity range of NPs.

Using Equation (1), we have calculated the adsorption energies of the functionalized silica NPs, and these are given in Table 1. From the data, it can be observed that the NP-NH_2_, followed by NP-OH, had the lowest desorption free energy from the P/W interface into the water phase, which indicates that these have the most polar surface, namely, the easiest to hydrate. On the other hand, the NP-C8 had by far the largest desorption energies, which indicates that their desorption from the P/W into the water phase is strongly disfavored, namely, that their surface is hydrophobic and that it is most difficult to hydrate of all the nanoparticles. The desorption free energy values for the NP-CN and NP-SH, Table 1, fall in between the above two extremes, but their magnitudes appear closer to those of the more polar NP-NH_2_ and NP-OH than to that of the NP-C8. Thus, from these calculated values of the interfacial desorption energies of the NPs from the P/W interface into the water phase, one can already rank the surface polarity of the NPs in the following order, from the most to the least polar: NP-NH_2_ > NP-OH > NP-SH ≈ NP-CN > NP-C8.

### 4.3. Determining the Surface Energy of Nanoparticles—The Polar and Disperse Components

The aim of this work was to demonstrate that the new method can quantitatively determine the surface energy of functionalized silica nanoparticles and its components from the interfacial immersion depth and the contact angles at the three-phase line. For this, there are several existing models, derived from the expression of the work of adhesion and Young’s equation:

(2) $$\gamma_{NP/W}+\gamma_{P/W}\cdot \cos\beta =\gamma_{NP/P}$$

where *β* is the contact angle of the nanoparticle at the interface,$\;\gamma_{P/W}$ is the polymer/water interfacial tension, $\gamma_{NP/W}$ is the nanoparticle/water interfacial tension, and $\gamma_{NP/P}$ is the interfacial energy of polymer/nanoparticle. $\gamma_{NP/P}$ can be written also as a function the work of adhesion between the polymer and nanoparticle $W_{NP/P}^{in\;water}$:

(3) $$\gamma_{NP/P}=\gamma_{P/W}+\gamma_{NP/W}-W_{NP/P}^{in\;water}$$

Combining Equations (2) and (3), the expression for the work of adhesion between the polymer and the nanoparticle is obtained:

(4) $$W_{NP/P}^{in\;water}=\gamma_{P/W}\left(1-\cos\beta \right)$$

The work of adhesion in the Owens-Wendt-Rabel-Kaelble (OWRK) [2] model is expanded in two contributions—a contribution for the polar interaction of both phases and the contribution for the dispersive van der Waals interaction:

(5) $$W_{NP/P}^{in\;water}=2\sqrt{\gamma_{P/W}^{d}\gamma_{NP/W}^{d}}+2\sqrt{\gamma_{P/W}^{p}\gamma_{NP/W}^{p}}$$

By combining Equations (4) and (5), we obtain the corresponding OWRK expression:

(6) $$\gamma_{P/W}\left(1+\cos\beta \right)=2\sqrt{\gamma_{P/W}^{d}\gamma_{NP/W}^{d}}+2\sqrt{\gamma_{P/W}^{p}\gamma_{NP/W}^{p}}$$

**Figure 4:**
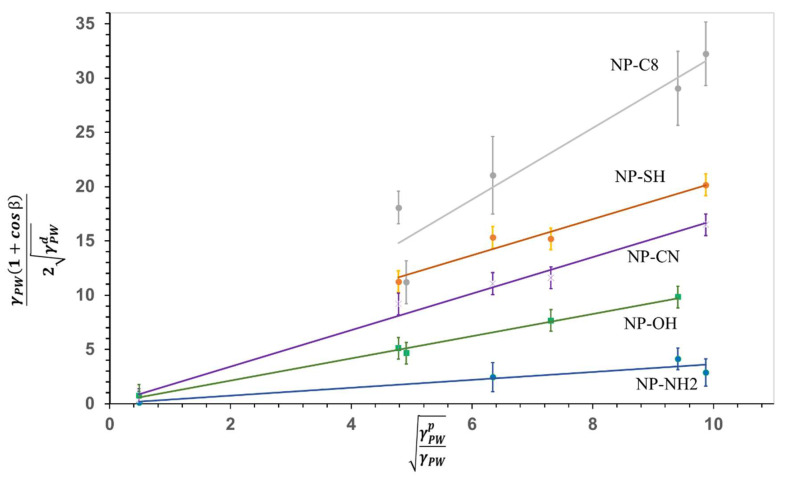
The linear OWRK equation constructed from the experimental data of the contact angles measured by the circular traces left by each nanoparticle, NP-NH2, NP-SH, NP-C8, and NP-OH, on the surfaces of different polymers. The data corresponding to NP-C8 were offset by −10, and the data of NP-SH by +3, for better visualization.

The linearized form of Equation (6) is obtained by dividing both sides of the equation by $\sqrt{\gamma_{NP/W}^{d}}$; the system of equations corresponding to β determined on different polymers with known surface energy and components $\gamma_{P/W}^{d}$ and $\gamma_{P/W}^{p}$(see Table S4), and can then be solved graphically, as depicted in Figure 4:

(7) $$\frac{\gamma_{P/W}\left(1+\cos\theta \right)}{2\sqrt{\gamma_{P/W}^{d}}}=\sqrt{\gamma_{NP/W}^{p}}\sqrt{\frac{\gamma_{P/W}^{p}}{\gamma_{P/W}^{d}}}+\sqrt{\gamma_{NP/W}^{d}}$$

By measuring the interfacial immersion depth and the contact angle β of each nanoparticle with at least three different polymer/water interfaces formed by polymerization of the Pickering emulsions, we calculated the total surface energy and its components $\gamma_{NP/W}^{p}$ and $\gamma_{NP/W}^{d}$ in water, by fitting the data to a linear equation, where $\sqrt{\gamma_{NP/W}^{p}}$ is the slope and $\sqrt{\gamma_{NP/W}^{d}}$ is the intercept, as shown in the Figure 4. The linear OWRK equation was constructed from the experimental data of the contact angle measured from the circular traces left by each nanoparticle, NP-NH2, NP-SH, NP-C8, and NP-OH. The experimental data of the circular hole diameters on the polymer colloidosomes left by the NPs and the calculated contact angles β are summarized in Table S5 in the Supplementary Material.

The obtained NP/W interfacial energies $\gamma_{NP/W}^{d}$ and $\gamma_{NP/W}^{p}$ are given in Table 2. From the data, the largest interfacial energy was between NP-C8 and water, as is to be expected from a hydrophobic surface. Next, the lowest interfacial energy was from NP-NH_2_, and then NP-OH, which suggests strong surface hydration and good dispersibility of the functionalized NPs in water. These data are supported by the values of the desorption free energies; see Table 1 and the discussion in the previous section. Additionally, except for NP-C8, the disperse interfacial energy components were significantly smaller than the polar components, which suggests that the disperse van der Waals interactions between nanoparticles and water are less substantial than other interactions, such as dipole-dipole and hydrogen bonding, which are both covered by the polar components. These results bring valuable insights into the interaction of the nanoparticle surface and water. The fact that the interfacial energy of NP-C8 with water is so large as compared to the other energies suggests the extreme sensitivity of the NanoTraPPED method.

Note that the work of adhesion of the NP to the polymer in water is different from that in air:

(8) $$W_{NP/P}^{in\;water}\neq \;W_{NP/P}^{in\;air}=2\sqrt{\gamma_{P}^{d}\gamma_{NP}^{d}}+2\sqrt{\gamma_{P}^{p}\gamma_{NP}^{p}}$$

where the subscripts P and N denote the interfaces of the polymer and nanoparticle in air.

The $\gamma_{NP/W}^{d}$ and $\gamma_{NP/W}^{p}$ values can easily be converted by using the rules [4] for combining the surface energy of nanoparticles in air, $\gamma_{NP}^{d}$ and $\gamma_{NP}^{p}:$

(9) $$\gamma_{NP}^{p}={\left(\sqrt{\gamma_{NP/W}^{p}}-\sqrt{\gamma_{NP/W}^{p}}\right)}^{2}$$

Based on the above formula, we have recalculated the values of the surface energies and their disperse and polar components, $\gamma_{NP}^{d}$ and $\gamma_{NP}^{p}$. They are given in Table 3. From the data, the total surface energy of the nanoparticles decreases in the series NP-NH_2_ > NP-C8 > NP-OH > NP-CN > NP-SH. The polarity of the nanoparticles as judged by the magnitude of the polar surface energy component $\gamma_{NP}^{p}$, decreases in the series NP-NH_2_ > NP-OH > NP-CN > NP-SH > NP-C8. In this case, one can consider that NP-NH_2_ were the most polar nanoparticles. In fact, this result of the polar component ranking of the surface energy is clearly supported by the desorption free energy data; see Table 1 and the previous discussion. However, when confronting these data with the theoretical dipole moment of each of the functional groups (see Table S3), one can see that the -CN have the largest dipole moment and consequently were expected to be the most polar in the series. However, this is not a clear indication, considering on the one hand the surface density of the functional groups, which can be different, but also the protonation of the -NH_2_ groups in the slightly acidic pH of water (pH = 5.6), which would make the later functionality more polar than -CN. On the other hand, the nanoparticles that interacted the most via disperse van der Waals forces $\gamma_{NP}^{d}$ were, in decreasing order: NP-C8 > NP-NH_2_ = NP-CN > NP-OH > NP-SH.

Next, it is instructive to compare the surface energy values obtained in this work, Table 2, with the literature values of silica NPs functionalized with the same functional groups. However, due to lack of available data on nanoparticles, we can only compare our values for NPs with the surface energy values from macroscopic silicon, silica, or glass surfaces modified with the same surface agents as those used in this work; see Table S7. When comparing our data with the literature values (Table 2) we observed that the X_p_ and X_d_ measured for NP-OH and NP-NH_2_ are in perfect agreement with the literature values obtained for the bare SiO_2_ and SiO_2_ surfaces functionalized with APTES; see Table S6. Further, the X_p_ and X_d_ of NP-SH are also very close to the values obtained for glass modified with MPTMS. Additionally, the X_d_ values for SiO_2_/OTS are larger than those for NP-C8 and smaller than those for SiO_2_/TESPN and NP-CN. At the same time, the total surface energy decreases in the order SiO_2_ (-OH) > SiO_2_/APTES (-NH_2_) > SiO_2_/MPTMS (-SH) ≈ SiO_2_/TESPN (-CN) > SiO_2_/OTS (-C8); see Table S7. That order is similar to the ranking we obtained (Table 2), with the exception that NP-NH_2_ has a higher surface energy than NP-OH. We attribute this latter observation to the fact that we worked in slightly acidic conditions, which led to the protonation of NP-NH_3_^+^. Overall, there seems to be good agreement between the literature values of the surface energy with polar and disperse contributions and the values obtained for NPs with the NanoTraPPED method.

### 4.4. Sources of Error, Limitations of the NanoTraPPED Method and Future Studies

To quantitatively estimate the uncertainties of the NanoTraPPED method, we have identified the main sources of systematic and random errors. Among the main sources of systematic errors that can arise when determining the interfacial immersion depths of nanoparticles are deviation from the monodispersity of the silica nanoparticles and interactions of the electron beam with the polymer. Although we tried as much as possible to synthesize monodisperse silica nanoparticles, deviations from ideal monodispersity are inherent; thus, we included this aspect in our calculations of errors. See a detailed discussion in SI. Additionally, we took extreme precautions to ensure that the interactions of the e-beam with the polymer in the SEM were eliminated, first by sputtering the sample Pt to dissipate the charge and the electron beam energy—i.e., landing energy was kept very low to 700 eV, by using a bias voltage of 1000 V—and by using the mirror and through-lens detectors. On the other hand, the sources of random error come mainly from how precisely we can determine the diameters of the circular traces from the SEM images. We included these errors in the calculations; see the discussion in the Supplementary Material. Among the random errors affecting the precision of the method is the oil droplet deformation, either due to shrinkage of the droplet during polymerization [43] or mechanical movement in the liquid phase during shearing and emulsion formation. Droplet deformation, especially during the liquid phase or gelation phase, can produce jamming effects, which cause “expelling out”—or the opposite effect of “burying in”—some of the NPs from the plane of the self-assembled interfacial layer. Other phenomena affecting the immersion depths of the NPs are the length scale of curvature perturbation of the interfacial droplet, which may very well set the lower limit to NPs sizes that can be analyzed by the NanoTraPPED method. Further, relaxation effects causing local “healing” of the circular traces and disappearance during the gelation phase could also be considered as factors affecting the precision. Although we took great care in the image analysis by selecting only the perfect circular traces on the largest colloidosomes, which were much larger than the NPs, the effects mentioned above affecting the precision of the method are not always discernable by post hoc rationalization and are accounted for during the SEM image analysis. Consequently, the precision of the method could be improved in the future by isolating various phenomena broadening the error when determining accurate diameters of the circular traces left by the nanoparticles. Further computational studies could shed some light into the processes taking place at the interface—for example, to understand how mechanical movement or interfacial shrinking and jamming causing curvature perturbations of the interface affect the NPs’ interfacial immersion depth [44,45].

## 5. Conclusions

In the current work we have introduced a new method, NanoTraPPED, for measurement of the total surface energy of nanoparticles bearing different functionalities by trapping them at various polymer water interfaces, via Pickering emulsion polymerization. For this we have used a series of functionalized silica NPs. We have shown that the OWRK model, which is typically applied in interfacial science for calculating the surface energy components of a macroscopic surface from the contact angles of sessile drops of at least three liquids differing in their polarity, can also be used for NPs trapped on polymer colloidosomes, on which they exhibit different interfacial immersion depths. From the contact angles of the functionalized NPs with various polymer colloidosomes we have calculated the total, disperse, and polar surface energy components of the NPs with respect to water and air. The OWRK model applied in NanoTraPPED was able to predict the most polar surface and rank their surface polarities quantitatively. In fact, the power of the model to rank the surface polarities according to the polar and disperse components of NPs was validated by an independent model, namely, the desorption free energy of NPs from the P/W interface into the water phase. Currently, we have demonstrated the viability of the NanoTraPPED method for measuring the surface energy of functionalized silica NPs with ≈500 nm diameters, but future work is needed to further explore the potential of the NanoTraPPED method for various particle sizes, to set the lower and the upper boundaries in terms of NP sizes.

We believe that knowledge of the polar and disperse surface energy components of NPs could help explain the bulk nanopowder behavior, with respect to its flowing ability, dispersibility in a medium, adhesion to surfaces, etc. In future, NanoTraPPED can be greatly expanded to use different surface energy models, such as acid-base, extended Fowkes, and OCG (van Oss, Choudhury, and Good). [4].

## Figures and Tables

**Table 1:** Desorption energy calculated from Equation (1) from the corresponding value of the contact angle *β* of the NP in the polymer, the interfacial energy *γ*_P/W_, and the radius of the nanoparticle.

NP	Polymer	γ_P/W_ (mJ/m^2^)	Radius (nm)	Cosβ	ΔG_desorbtion_ (KJ/mol)
NP-OH	PSty	35.48	250.34	−0.65	849.8
	PMM	20.82		−0.54	850.6
	PDAEMA	3.52		−0.28	354.7
	PBM	41.07		−0.67	860.4
	PVAc	26.47		−0.64	677.7
NP-C8	PSty	35.48	254.72	0.39	13,881.7
	PBM	41.07		0.79	26,915.7
	PtBA	1.05		0.43	438.9
	PVAc	26.47		0.64	14,564.2
	PEM	32.03		0.50	14,761.1
NP-CN	PMM	20.82	252.50	−0.31	1980.2
	PDAEMA	3.52		−0.66	82.1
	PBM	41.07		−0.41	2836.5
	PtBA	1.05		−0.49	54.7
	PEM	32.03		−0.41	2212.1
NP-SH	PMM	20.82	249.67	−0.28	2138.4
	PBM	41.07		−0.47	2234.2
	PtBA	1.05		−0.43	66.2
	PEM	32.03		−0.39	2340.1
NP-NH_2_	PSty	35.48	249.43	−0.85	148.9
	PDAEMA	3.52		−0.88	10.5
	PBM	41.07		−0.89	101.8
	PEM	32.03		−0.90	65.7

**Table 2:** Interfacial energy and its disperse $\gamma_{NP/W}^{d}$ and polar components; $\gamma_{NP/W}^{p}$ of the silica nanoparticles with water, bearing different surface functional groups. The errors in the obtained values of the surface energies are given in Table S6.

Nanoparticle	γ^d^_NP/water_ (mN/m)	γ^p^_NP/water_ (mN/m)	γ_NP/water_ (mN/m)
NP-OH	0.00	1.06	1.06
NP-C8	111.65	9.79	121.44
NP-CN	0.00	2.77	2.77
NP-NH_2_	0.00	0.14	0.14
NP-SH	0.21	3.10	3.10

**Table 3:** Surface energy (interfacial energy of the nanoparticles with air) γ_NP_ and its disperse $\gamma_{NP}^{d}$ and polar components $\gamma_{NP}^{p}$ for the silica nanoparticles with water, bearing different surface functional groups. X_d_ and X_p_ are the corresponding fractions of the disperse and polar components of the total surface energy.

Nanoparticle	γ^d^_NP_ (mN/m)	X_d_	γ^p^_NP_ (mN/m)	X_p_	γ_NP_ (mN/m)
NP-OH	25.40	0.43	33.92	0.57	59.32
NP-C8	30.54	0.69	13.89	10.31	44.43
NP-CN	25.40	0.49	26.94	0.51	52.34
NP-NH_2_	25.40	0.38	41.96	0.62	67.36
NP-SH	20.96	0.44	26.60	0.56	47.56

## Data Availability

The data generated in this study is publicly available in an open access repository Open Science Framework (OSF) repository at DOI 10.17605/OSF.IO/TYM28.

## Supplementary Materials

The following are available online at https://www.mdpi.com/article/10.3390/nano11123200/s1: Description of nanoparticle synthesis Scheme S1 and functionalization Figure S2. Characterization results, such as SEM images of silica NPs, Figures S1 & S3; tables with zeta potentials and NP diameters Table S1; FTIR spectra and interpretations of functionalized NPs Figure S4. EDX spectra of the functionalized NPs, Figure S5 and the photograph of the ninhydrine test Figure S6. Chemical structures of monomers used in Pickering emulsions, Figure S7; table with recipes used for the preparation and polymerization of Pickering emulsions, Table S2; optical microscopy images and SEM images of the obtained colloidosomes showing traces of NPs on their surfaces, Figures S8, S9, S10, S11 & S12; dipole moment of some functional groups, Table S3; a table, Table S4, and a graph, Figure S13, showing the ranking in polarity of the polymers used for NanoTraPPED; a table with the hole diameters left by the functionalized NPs on various colloidosomes, Table S5; discussion of error analysis and formulas used for error propagation. Polar and disperse contributions of the surface energies and associated fitting errors in slope and intercept for the NPs studied, Table S6. Literature values of the surface energies of the polar and disperse components for surfaces with the relevant functional groups, Table S7.
