# Supplementary material for: NanoTraPPED—A New Method for Determining the Surface Energy of Nanoparticles via Pickering Emulsion Polymerization
Source: Nanomaterials (Basel). 2021 Nov 25;11(12):3200. doi: 10.3390/nano11123200 (PMC8709214; doi:10.3390/nano11123200)
Supplement: Supplementary file 1 [file nanomaterials-11-03200-s001.zip › nanomaterials-1459189-supplementary.pdf]

Supplementary Information

NanoTraPPED - a New Method for Determining the Surface Energy of Nanoparticles via Pickering Emulsion Polymerization

Andrei Honciuc[†]*, Oana-Iuliana Negru[†]

[†] *"PetruPoni" Institute of Macromolecular Chemistry, Electroactive Polymers and Plasmochemistry Laboratory, Aleea Gr. GhicaVoda 41A, Iasi, 700487, Romania*

Experimental

Synthesis of silica nanoparticles

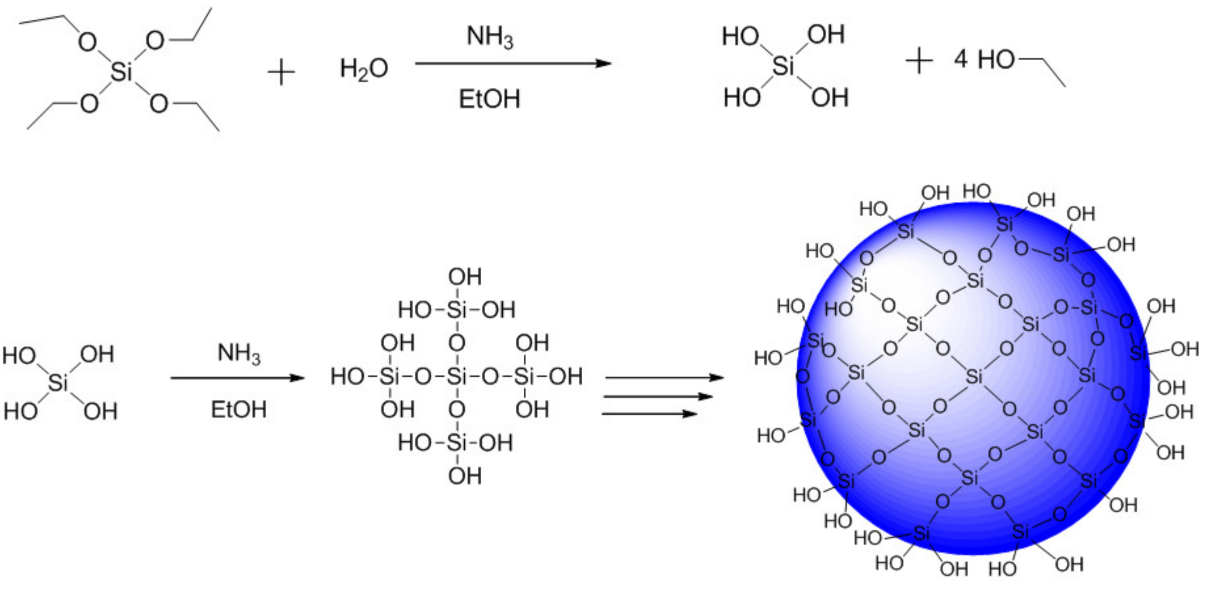

Scheme S1. Reaction scheme of synthesis of silica nanoparticles from TEOS.

Synthesis of silica nanoparticles:

Monodisperse SiO_2 spheres were prepared using a modified procedure originally described by Stöber et al. In Figure S2 shows the micromorphology of silica nanoparticles (drying of an aqueous particles suspension on the surface of the aluminium sample holder), which

demonstrating that uniform and well-formed spherical nanoparticles with a size of approximately 500±7 nm.

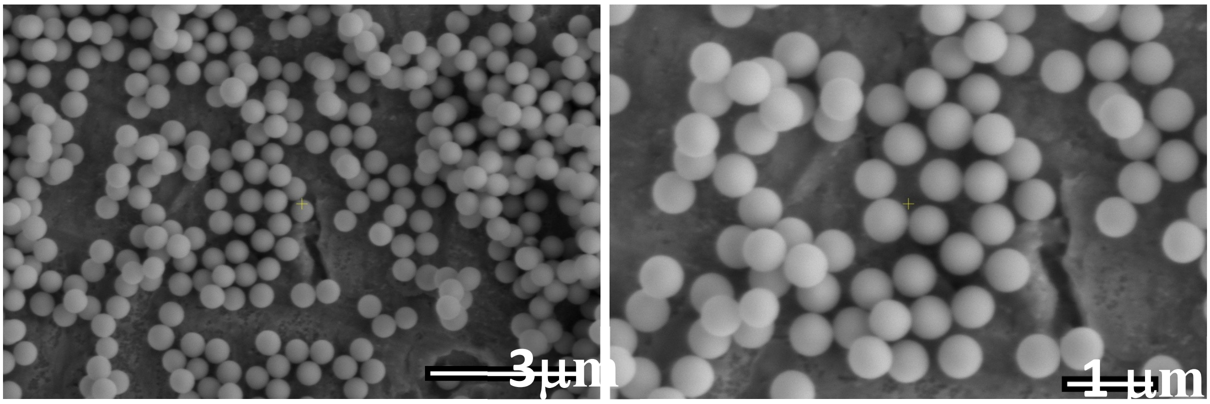

Figure S1. SEM images of silica nanoparticles at two different magnifications.

Surface modification of the silica nanoparticles

By modifying the surface of the nanoparticles with different silane coupling agents, then the hydroxyl groups can be replaced by groups with variable polarities, to achieve the transformation of the silica surface from hydrophilic to hydrophobic. The reagents used for the various modifications are listed in Figure S3. SEM micrographs of the prepared particles are shown in Figure 3, which demonstrates that the modified particles are spherical with uniform size and shape.

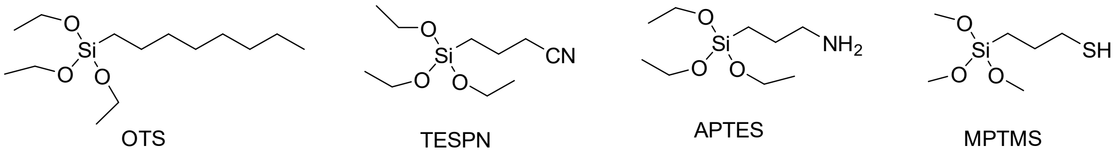

Figure S2. Various functional groups used for the surface modification of silica nanoparticles.

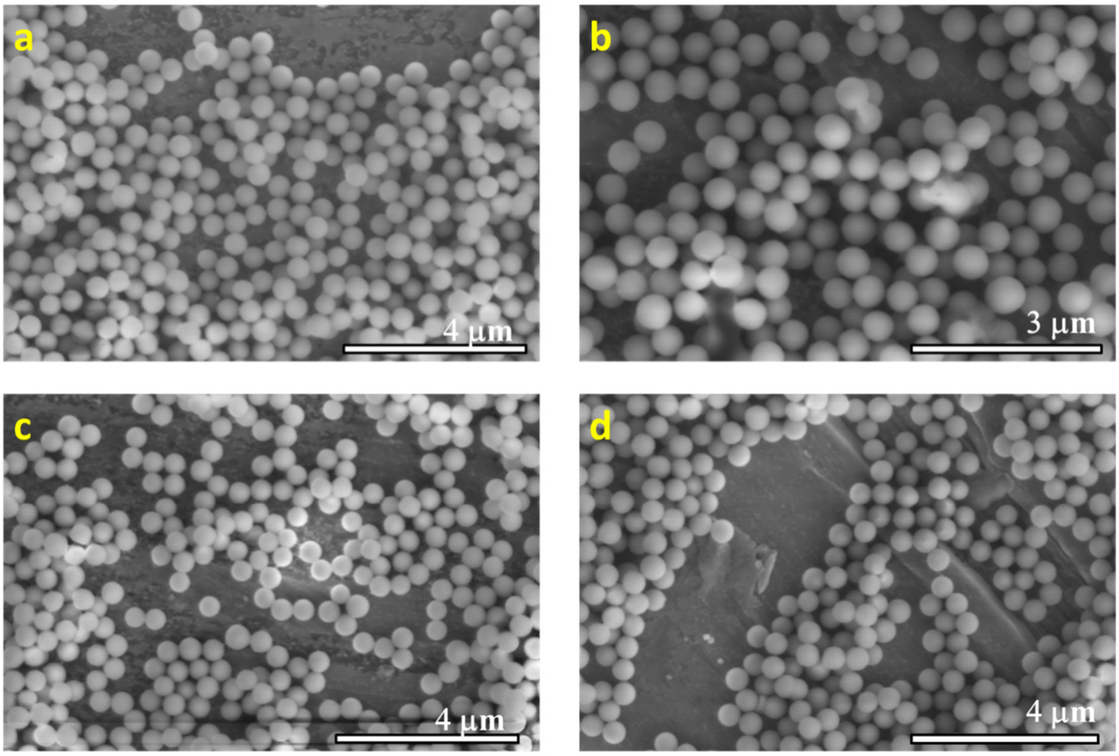

Figure S3. Representative SEM image of silica nanoparticles modified:NP-C8 (a), NP-CN (b), NP-NH_2 (c) and NP-SH (d).

The first method used to control the success of surface modification was to measure the zeta potential of the particles. All samples were prepared with the same concentration for a better comparation. Results are shown in the Table S1.

Table S1. Unmodified and the modified the silica nanoparticles were measured with SEMandZetaSizer to ζ-potential.

Functional group at the surface	Diameter / SEM [nm]	ζ-potential [mV]
NP-OH (starting material NPs)	500±7	-49.7 ± 1.4
NP-C8	509±5	-47.4 ± 0.8
NP-CN	505±5	-45.5 ± 0.5
NP-NH_2	500±5	-15.5 ± 0.8
NP-SH	500±4	-51.7 ± 0.2

Figure S4 shows the FTIR spectrum of pristine silica NPs and functionalized silica NPs. The silica NPs are composed of mainly Si-O-Si networks and include –OH and –OEt groups as additional functional groups as well as EtOH and water as a residual component. Unmodified silica nanoparticles showed strong signals at 1060 $cm^{-1}$and 796 cm^{-1} associated with the asymmetric and symmetric stretching of Si-O-Si, respectively. The broad band at 950 cm^{-1} was due to the Si-O in plane stretching vibrations in the Si-OH group, while the peaks at 3427 and 1631 cm^{-1} were attributed to the overlapped band of O-H stretching of physically adsorbed water and the Si-OH stretching of the silanol group and the O-H deformation vibration of the adsorbed water, respectively. The vibration bands at 2966 – 2852 cm^{-1} relate to the symmetric and asymmetric stretching of $–CH_2$ from Si-O-Et.

NP-C8: In the region 2800 – 3000 cm^{-1} an increased in bands intensities is observed. It indicates that the alkyl chain has been grafted onto NPs surface. Moreover, the intensity of the band at 950 cm^{-1}decreased, this indicated that some silanol groups were modified.

NP-CN: The peak at 2247 cm^{-1} corresponds to the nitrile group. Moreover, the intensity of the band at 950 cm^{-1}decreased and in the region 2800 – 3000 cm^{-1} an increased in bands intensities can be observed. These indicate that the NPs surface was grafted with the nitrile-organo-alkoxyl group on the silica surface through silanol groups.

NP-NH_2: Primary amines typically display two weak absorption bands at 3500 cm^{-1} and the other near 3400 cm^{-1}. These bands represent the free asymmetrical and symmetrical N-H stretching modes. Unfortunately, their absorption peaks overlap with the peaks of the O-H stretch in water. Increases of relative intensity of the vibrations bands corresponding of CH_2 and a decrease in the band intensity from 950 cm^{-1} indicate that the NPs surface was grafted of the amino-organoalkoxyl groups on the surface of silica.

NP-SH: The peaks corresponding to the –SH group (a signal between 2550 and 2600 cm^{-1}) could not be clearly observed in the spectrum, owing to the strong presence of vibrationpeaksof

the silica in this region. But nevertheless, the intensity of the band at 950 cm^{-1}decreased, this indicated that some silanol groups were modified, and the region 2800 – 3000 cm^{-1} an increased in bands intensities is observed. This indicates that the alkyl chain were grafted onto NPs surface.

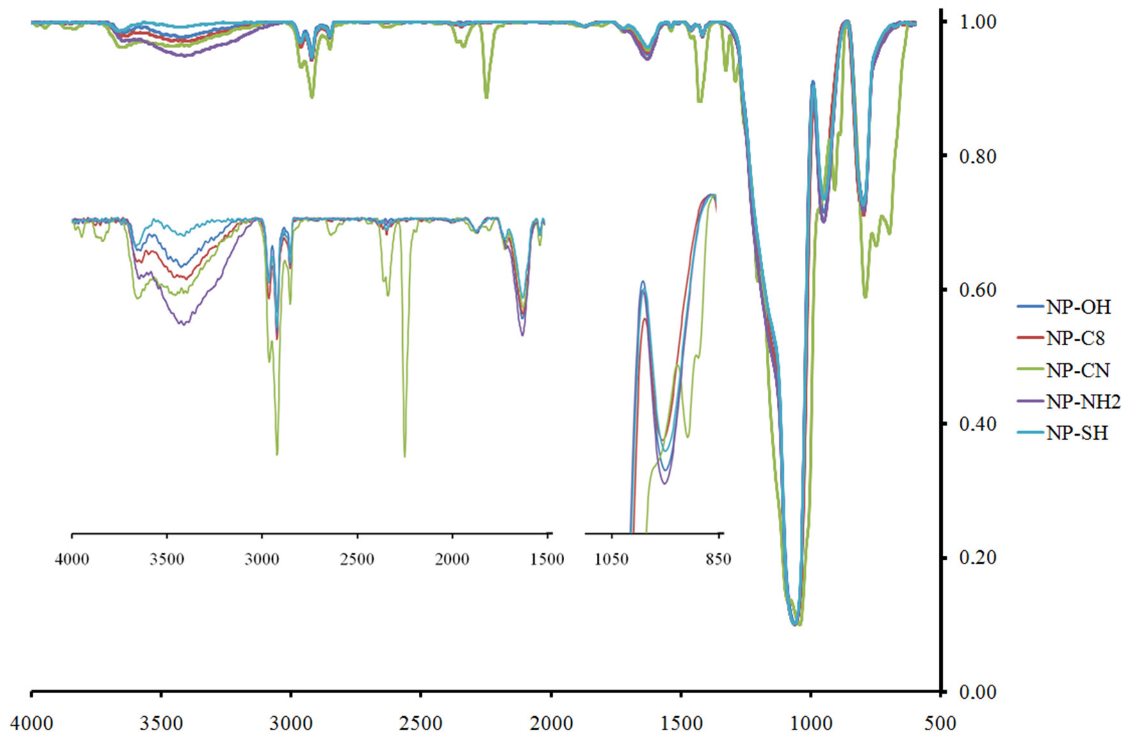

Figure S4. FTIR of starting NP-OH and after functionalization NP-C8, NP-CN, NP-NH2, NP-SH.

Figure S4a shows the results of the EDX analysis of starting NP-OH and after functionalization NP-C8, NP-CN, NP-SH. The EDX results can be judged in qualitative terms. In the NP-CN sample Nitrogen is present, proving that the -CN functionality was successful; in the NP-S sample the Sulfur is present proving successful functionalization of the nanoparticles. In the starting NP-OH Oxygen is present in high concentration, Carbon is present probably due to contamination and from atmosphere. For the NP-NH_2 sample the EDX results were not conclusive therefore, to prove the presence of the amine functionality we have performed the ninhydrine test. The ninhydrine test results in Figure 4b prove that the -NH_2 functionality is indeed present on the surface of the nanoparticles.

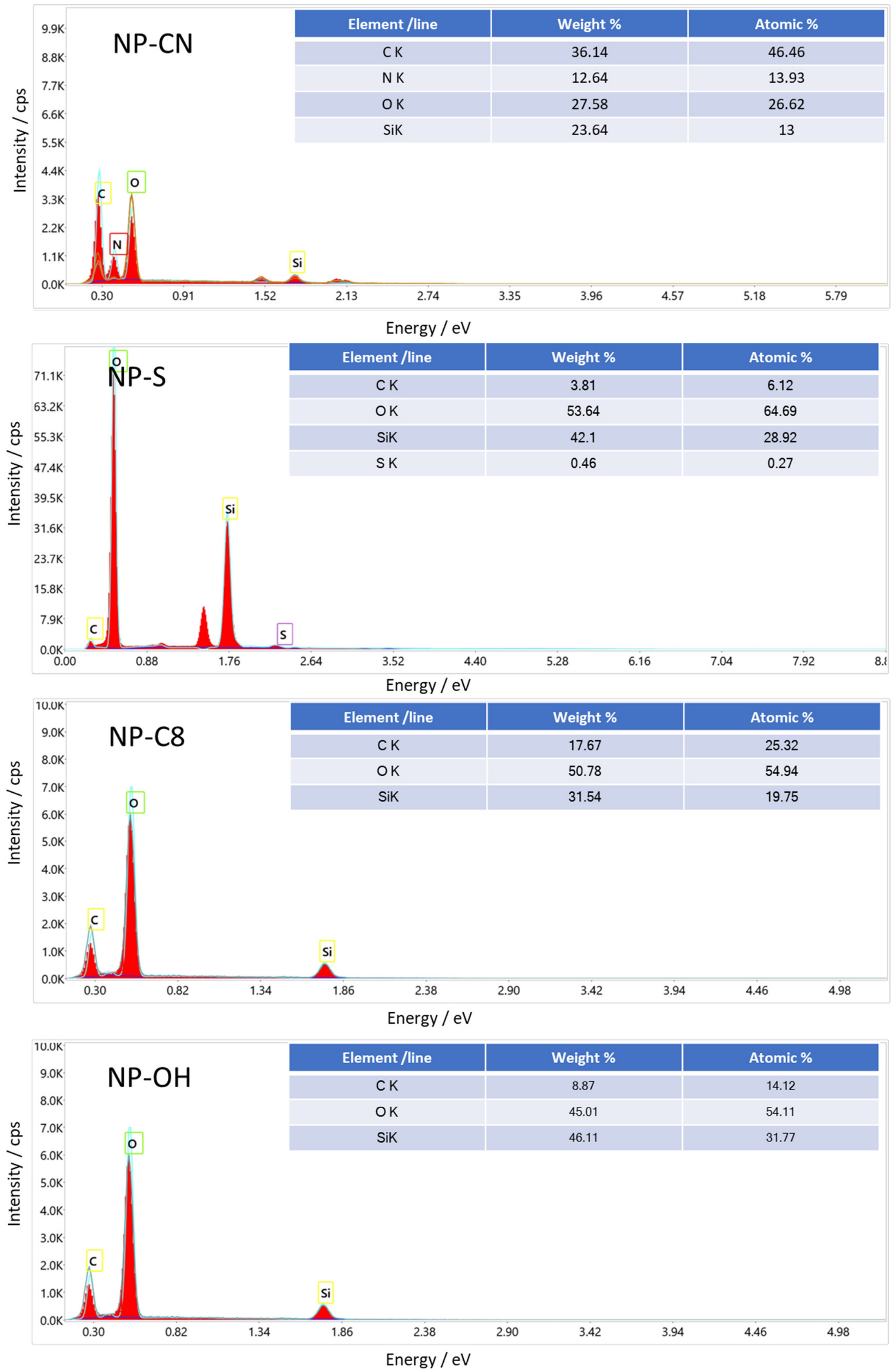

NP-CN

Element /line	Weight %	Atomic %
C K	36.14	46.46
N K	12.64	13.93
O K	27.58	26.62
SiK	23.64	13

NP-S

Element /line	Weight %	Atomic %
C K	3.81	6.12
O K	53.64	64.69
SiK	42.1	28.92
S K	0.46	0.27

NP-C8

Element /line	Weight %	Atomic %
C K	17.67	25.32
O K	50.78	54.94
SiK	31.54	19.75

NP-OH

Element /line	Weight %	Atomic %
C K	8.87	14.12
O K	45.01	54.11
SiK	46.11	31.77

Figure S5. EDX analysis of starting NP-OH and after functionalization NP-C8, NP-CN, and NP-SH.

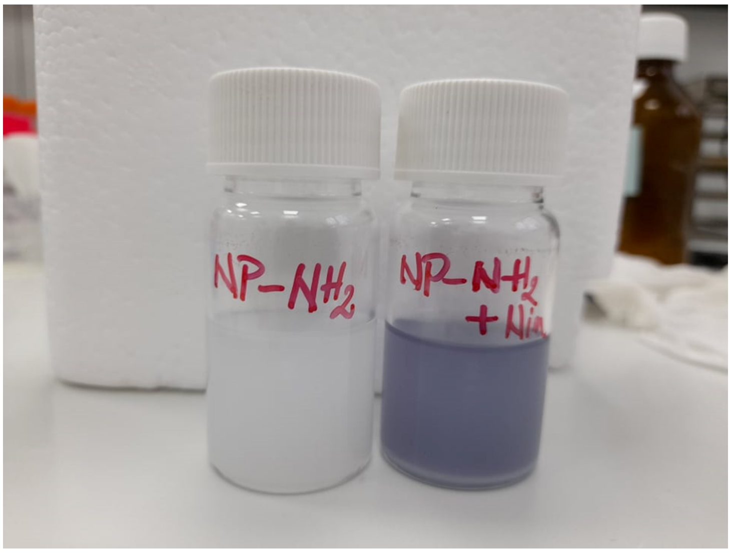

Figure S6. Photograph with the NP-NH_2 particles suspended in water with (blue) and without ninhydrine (white), proving that the -NH_2 functionality is present.

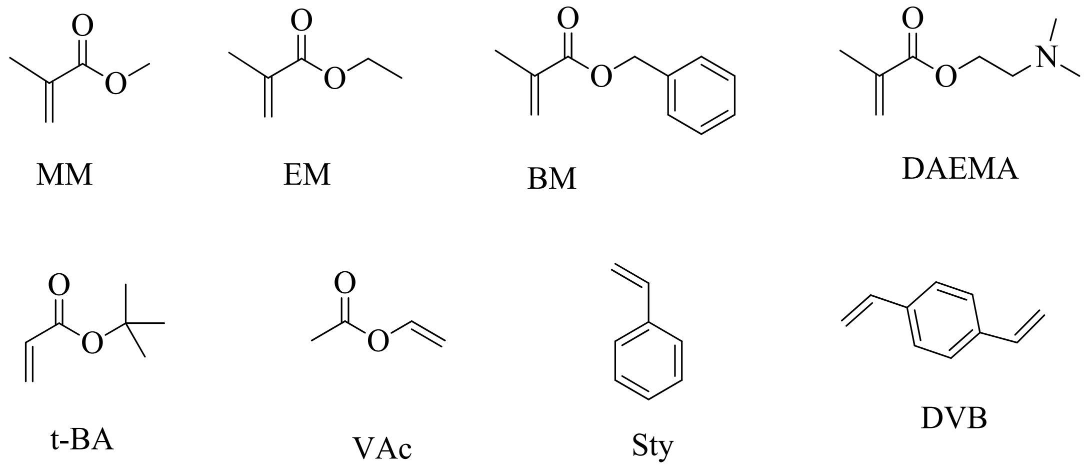

Figure S7. Vinyl bearing monomers used for the Pickering emulsion formation and polymerization.

Table S2. A summary of the conditions used in the colloidosome synthesis.

Functional Group	Monomer/ [mL]	DVB [mL]	BME [mg]	H_2O [mL]	NP [mg/mL]	Sonication [s]	Amplitude [%]
NP-OH	Sty - 1	0.1	20	12	5	15	30
	DAEMA - 1	0.1	20	12	5	15	30
	BM - 1	0.1	20	12	5	15	30
	MM -1	0.1	20	12	5	15	30
	VAc - 1	0.1	20	12	5	15	30

NP-C8	Sty - 1	0.1	20	12	5	15	30
	BM - 1	0.1	20	12	5	15	30
	t-BA - 1	0.1	20	12	5	15	30
	VAc - 1	0.1	20	12	5	15	30
	EM - 1	0.1	20	12	5	15	30
NP-CN	MM - 1	0.1	20	12	5	15	30
	DAEMA -1	0.1	20	12	5	15	1000 (Vortex)
	BM - 1	0.1	20	12	5	15	30
	t-BA - 1	0.1	20	12	5	15	30
	EM - 1	0.1	20	12	5	15	30
$NP-NH_2$	Sty - 1	0.1	20	12	5	15	30
	DAEMA - 1	0.1	20	12	5	15	30
	BM - 1	0.1	20	12	5	15	30
	EM - 1	0.1	20	12	5	15	30
NP-SH	MM - 1	0.1	20	12	5	15	30
	BM - 1	0.1	20	12	5	15	30
	t-BA - 1	0.1	20	12	5	15	30
	EM - 1	0.1	20	12	5	15	30

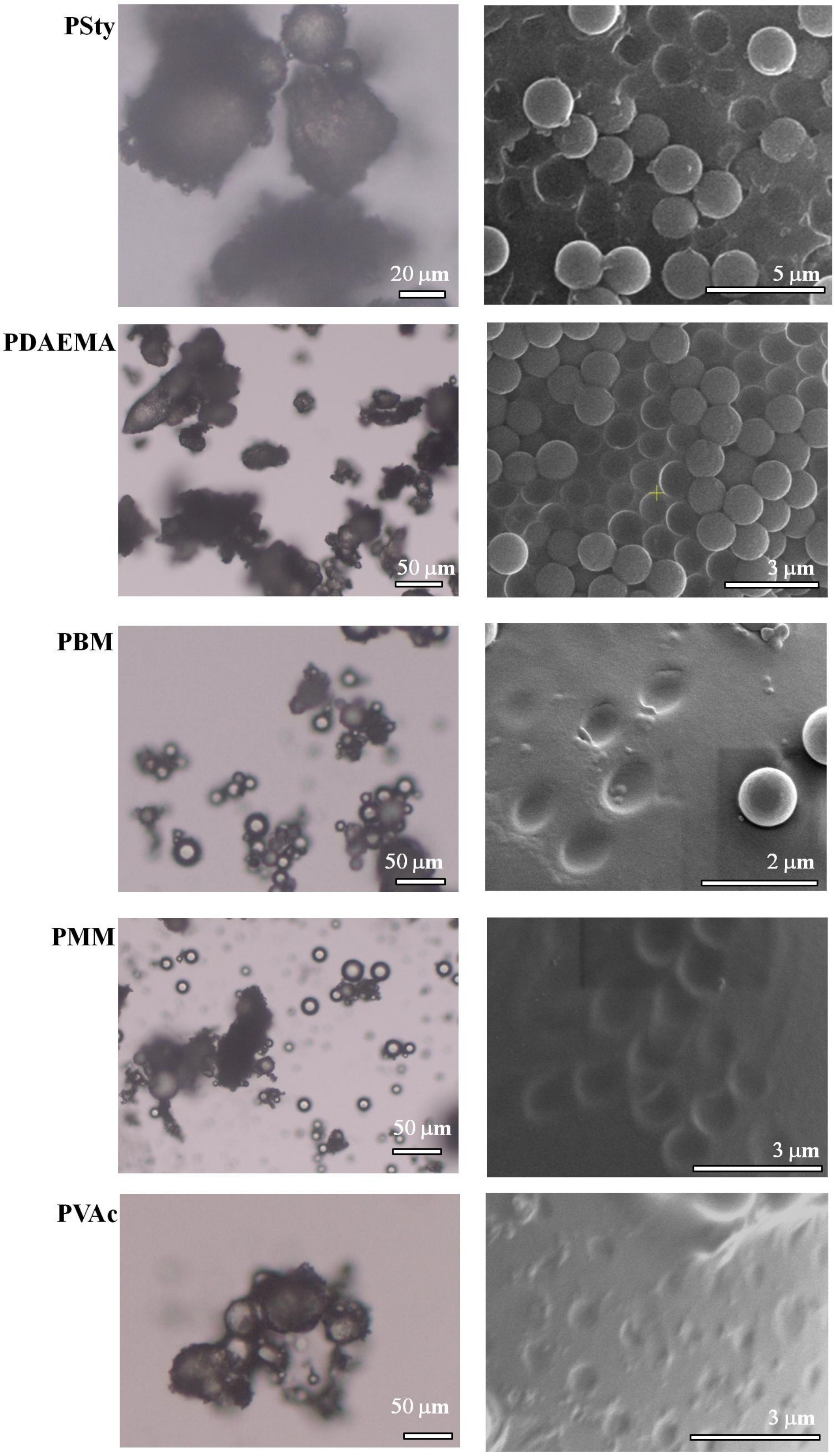

Figure S8. Left: optical microscope images of the polymerized o/w Pickering emulsions, consisting of spherical microparticles - colloidosomes; Right: SEM images of the surface of the colloidosomes showing NP-OH nanoparticles and circular traces left after their removal by sonication.

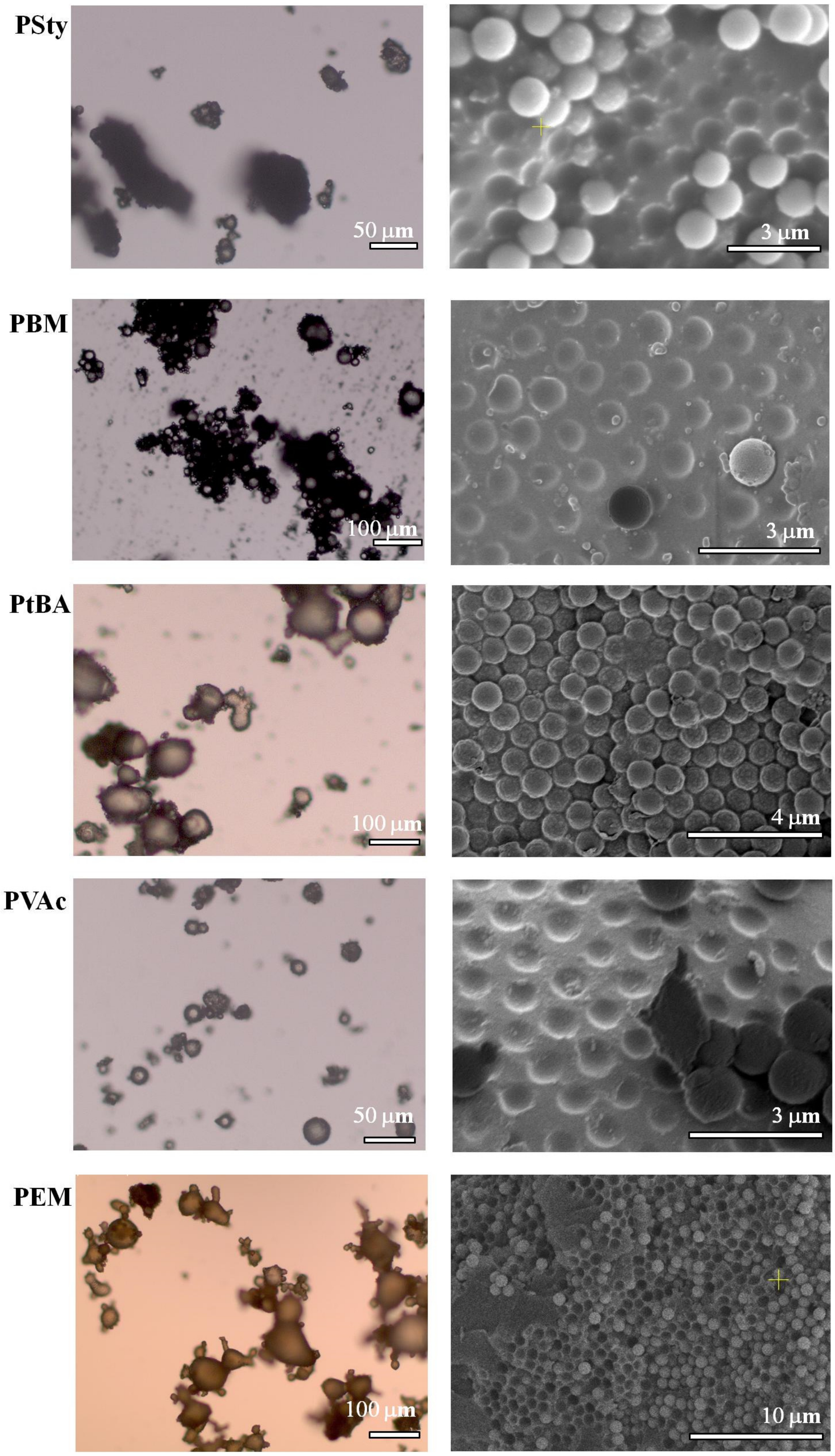

Figure S9. Left: optical microscope images of the polymerized o/w Pickering emulsions, consisting of spherical microparticles - colloidosomes; Right: SEM images of the surface of the colloidosomes showing NP-C8 nanoparticles and circular traces left after their removal by sonication.

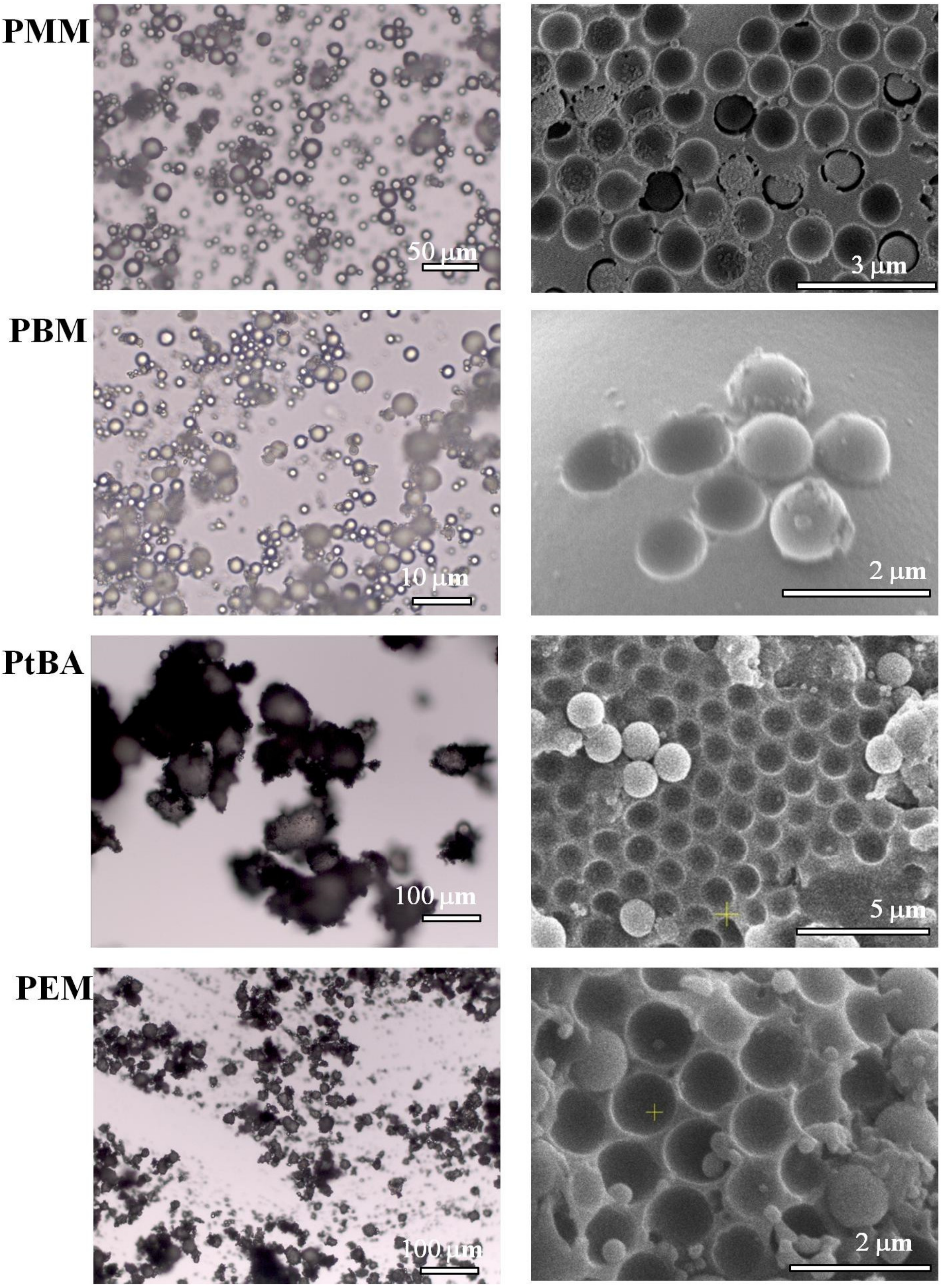

Figure S10. Left: optical microscope images of the polymerized o/w Pickering emulsions, consisting of spherical microparticles - colloidosomes; Right: SEM images of the surface of the colloidosomes showing NP-CN nanoparticles and circular traces left after their removal by sonication.

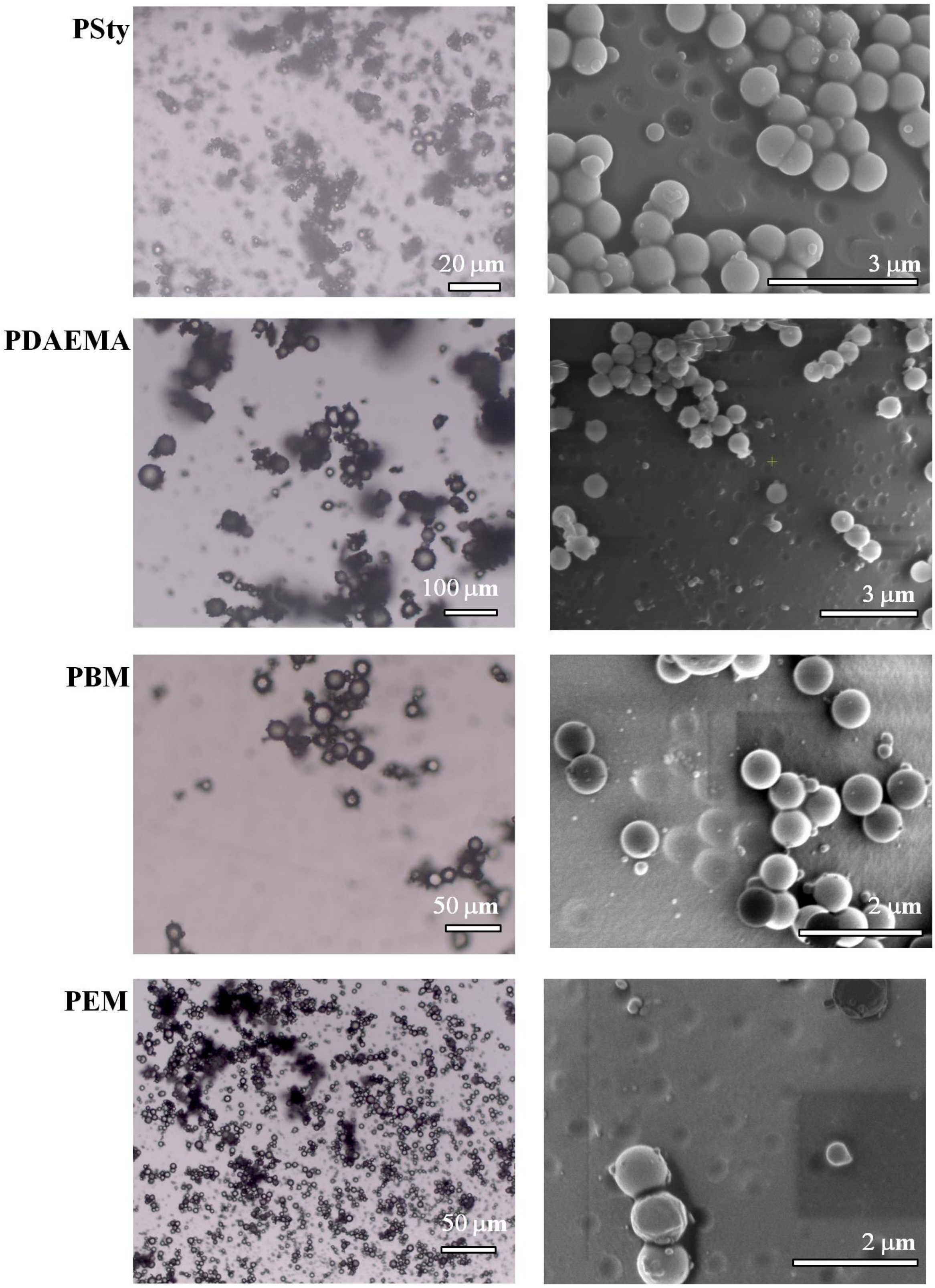

Figure S11. Left: optical microscope images of the polymerized o/w Pickering emulsions, consisting of spherical microparticles - colloidosomes; Right: SEM images of the surface of the colloidosomes showing NP-NH2 nanoparticles and circular traces left after their removal by sonication.

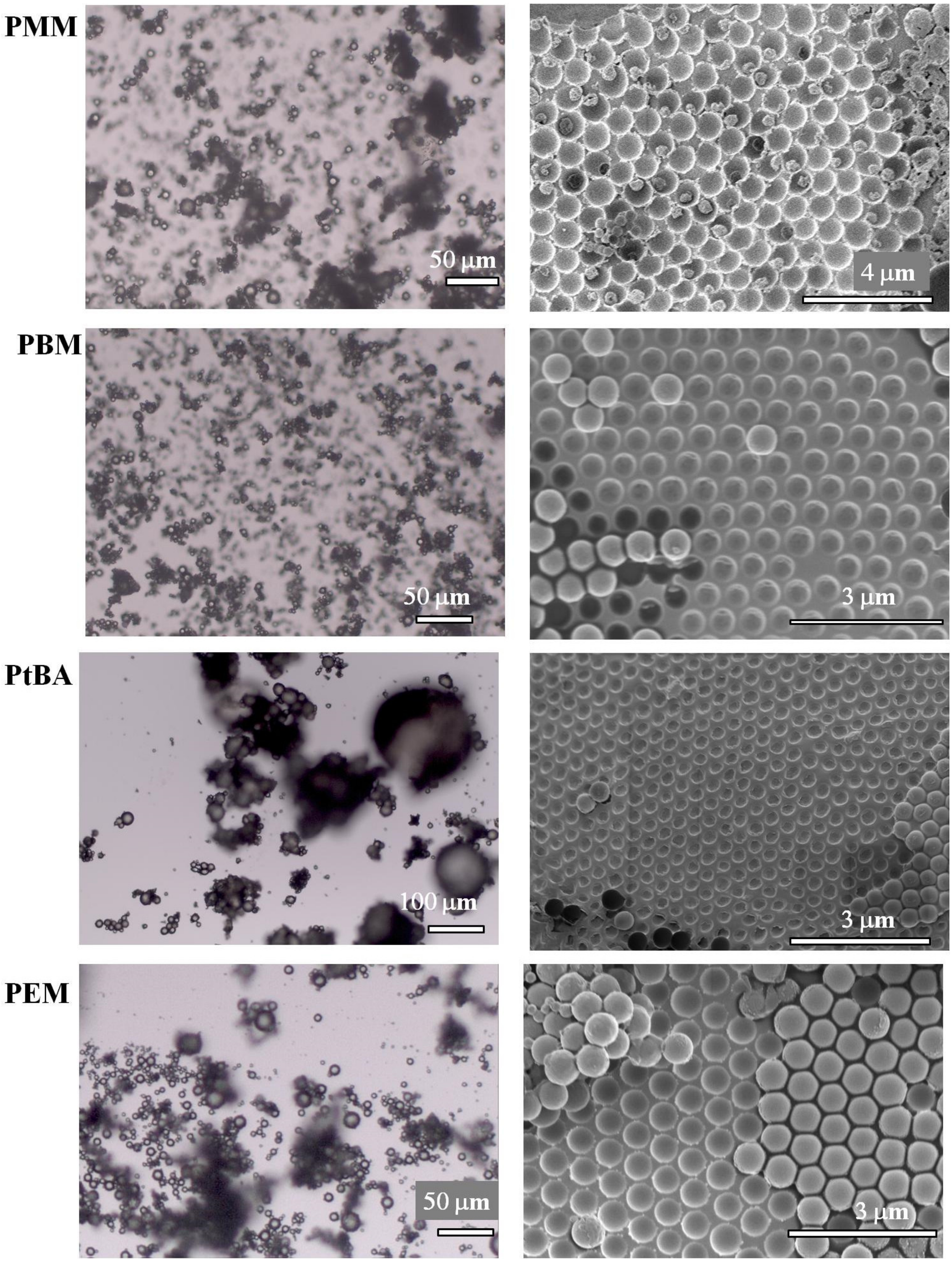

Figure S12. Left: optical microscope images of the polymerized o/w Pickering emulsions, consisting of spherical microparticles - colloidosomes; Right: SEM images of the surface of the colloidosomes showing NP-SH nanoparticles and circular traces left after their removal by sonication.

Table S3. Ranking of the polarity of the functional groups by their dipole moment according to literature.

Functional group	Dipole moment
-OH	1.69[1], 1.65[2]
-NH2	0.99[1], 1.2[2]
-SH	1.39[1]
-C8	0.4[2]
Carboxy	2.7[2]
- CN (Cyano)	3.4[2]
$-NO_2$ (Nitro)	3.1[2]
-Cl	2.04[1], 1.7[2]

To determine the surface energy with the polar and disperse components, the contact angles of five different solvents per polymer have been measured, see Table S4; the contact angle measurements have been repeated three times. The average contact angle values were used to calculate the surface energy of the polymers using the OWRK model.

Tabel S4. Surface energies and the polar and disperse components at the polymer/water (P/w) and polymer/air (P) interfaces of the polymers (with the same composition as given in Tabel S2) used in the current work,weredetermined by the OWRK method.

Polymer	γ^p_P (mN/m)	γ^d_P (mN/m)	γ_P (mN/m)	$\sqrt{\gamma_P^p / \gamma_P^d}$ (mN/m)	$\gamma^p_{P/w}$ (mN/m)	$\gamma^d_{P/w}$ (mN/m)	$\gamma_{P/w}$ (mN/m)
PSty	0.87	32.59	33.46	0.16	35.08	0.40	35.48
PMM	5.45	32.47	37.92	0.41	20.44	0.38	20.82
PDAEMA	36.42	11.5	47.92	1.78	0.67	2.85	3.52
PBM	0.34	40.85	41.19	0.09	39.35	1.72	41.07
PtBA	0.03	37.61	37.64	0.03	44.66	1.11	45.76
PVAc	3.29	37.28	40.57	0.30	25.42	1.05	26.47
PEM	1.5	31.92	33.42	0.22	31.71	0.33	32.03

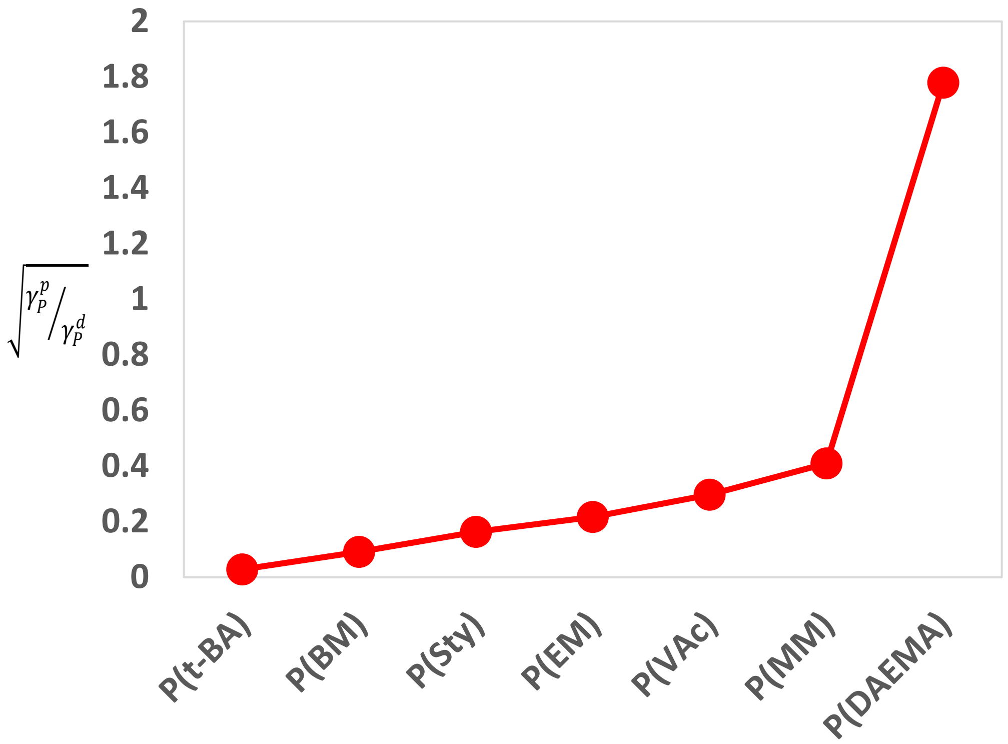

Figure S13. The ranking of the polymer surface polarity by taking the ratio of the polar to disperse component $\sqrt{\gamma_P^p / \gamma_P^d}$.

Table S5. Summary of all hole diameter, contact angle with the polymer.

Functional Group	Polymer	Hole diameter [nm]	β °
NP-OH	P(Sty)	380 ± 5	130.6 ± 1.7
	P(DAEMA)	480 ± 5	106.5 ± 4.2
	P(BM)	370 ± 5	132.3 ± 1.6
	P(MM)	420 ± 5	122.9 ± 2.1
	P(VAc)	385 ± 4	129.7 ± 1.4
NP-C8	P(Sty)	470 ± 3	112.7 ± 1.8
	P(BM)	310 ± 3	142.5 ± 8.1
	P(t-BA)	460 ± 6	115.4 ± 3.2
	P(VAc)	390 ± 6	130.0 ± 2.1
	P(EM)	440 ± 6	120.2 ± 2.8
NP-CN	P(MM)	480 ± 3	108.1 ± 2.9
	P(DAEMA)	380 ± 5	131.2 ± 1.4

	P(BM)	460 ± 4	114.4 ± 2.2
	P(t-BA)	440 ± 5	119.4 ± 1.8
	P(EM)	460 ± 5	114.4 ± 2.2
$NP\text{-}NH_2$	P(Sty)	260 ± 5	148.6 ± 1.4
	P(DAEMA)	240 ± 4	151.2 ± 1.1
	P(BM)	340 ± 6	137.0 ± 1.7
	P(EM)	220 ± 5	153.8 ± 1.3
NP-SH	P(MM)	480 ± 6	106.0 ± 5.2
	P(BM)	440 ± 4	118.2 ± 1.9
	P(t-BA)	450 ± 5	115.7 ± 2.6
	P(EM)	460 ± 4	112.9 ± 2.6

Table S6. Polar and disperse contribution of the surface energies and associated fitting errors obtained from the error in slope (polar component) and intercept (disperse component).

**Nanoparticle**	$\gamma^{d}_{NP/water}$(mN/m) (intercept^2)	Error in intercept	$\gamma^{p}_{NP/water}$(mN/m) (slope^2)	Error slope
NP-OH	0.00	2.32	1.06	0.29
NP-C8	111.65	3.24	9.79	0.40
NP-CN	0.00	1.95	2.77	0.29
$NP\text{-}NH_2$	0.00	1.03	0.14	0.12
NP-SH	0.21	4.41	3.10	0.53

Error analysis

To correctly interpret the sources of errors in NanoTraPPED and quantitatively calculate them we must consider the random errors, or the uncertainties in the measurement and the systematic errors, the errors that have the main source the physical nature of the nanoparticles and their deviation from monodispersity.

Random errors: these come mostly from the uncertainty in measuring the diameter of the circular traces from the SEM images. Such that, when measuring the diameter of the circular traces L, an error δL is produced, and the radius of the silica nanoparticles R an error δR is produced. Because the contact angle is calculated from L & R according to the formula:

$$\theta = \sin^{-1}\frac{L}{R} \quad \text{(S1)}$$

the error in θ must be propagated.

To find out the uncertainty in the contact angle $\theta \pm \delta\theta$ as a function of the uncertainties in the length of the circular traces left by the nanoparticles $L \pm \delta L$ and the uncertainties in the radius of the nanoparticle $R \pm \delta R$ we apply the general formula for the propagation of error:

$$\delta\theta = \sqrt{\left(\frac{d\sin^{-1}\frac{L}{R}}{dL}\delta L\right)^2 + \left(\frac{d\sin^{-1}\frac{L}{R}}{dR}\delta R\right)^2} \quad \text{(S2)}$$

Which, after calculating the partial derivatives results in the final expression:

$$\delta\theta = \sqrt{\left(\frac{1}{\sqrt{1-\left(\frac{L}{R}\right)^2}}\frac{1}{R}\delta L\right)^2 + \left(\frac{1}{\sqrt{1-\left(\frac{L}{R}\right)^2}}\frac{1}{R^2}\delta R\right)^2} \quad \text{(S3)}$$

Systematic errors: we have reasoned that the deviations of nanoparticle sizes away from monodispersity should be considered also as a systematic error, $\delta R = \delta R_{systematic}$, because it affects the precision with which L can be determined, which comes in addition to the random measurement error $\delta L = \delta L_{random}$. Thus, we considered the following formula, whereas δL_{random} and $\delta R_{systematic}$ can be combined in quadrature:[3]

$$\delta L_{total} = \sqrt{\delta L_{random}{}^2 + \delta R_{systematic}{}^2} \quad \text{(S4)}$$

Total error: thus, the total error in the NanoTraPPED including systematic and random error can be calculated by including the expression of δL_{total} in equation S3, with the final expression given by:

$$\delta\theta_{total} = \sqrt{\left(\frac{1}{\sqrt{1-\left(\frac{L}{R}\right)^2}}\frac{1}{R}\delta L_{total}\right)^2 + \left(\frac{1}{\sqrt{1-\left(\frac{L}{R}\right)^2}}\frac{1}{R^2}\delta R\right)^2} \quad (S5)$$

Based on the above, we have propagated the errors and determined that the main source of systematic errors, namely the deviation from an ideal monodispersity of the NPs will propagate significant errors in the contact angle. For example, a standard error of ± 4 nm in NPs diameter vs. a ±10 nm in NPs diameter will propagate an error in the contact angle between 1.5° to 4°, also depending on the magnitude of other parameters, see equation S5.

Correspondingly the propagated error $\delta\cos\theta$ in the OWRK equation is:

$$\delta\cos\theta = \sqrt{(|\sin\theta|\delta\theta_{total})^2}$$

Table S7. Literature values of the surface energies with the polar and disperse components (absolute and fractional values) for surfaces the relevant functional groups.

**Functional surface**	γ^d_{NP} (mN/m)	X_d	γ^p_{NP} (mN/m)	X_p	γ_{NP} (mN/m)	Substrate/Surface modifying agent	Measurement method
-OH	29.4	0.4	44.1	0.6	73.5[4]	SiO_2	Sessile drop
-C8	28	0.90	3	0.097	31[5]	SiO_2/OTS	Sessile drop
-CN	9	0.22	31	0.775	40[6]	SiO_2/TESPN	Sessile drop
$-NH_2$	21.81	0.38	35.59	0.62	57.4[4]	SiO_2/APTES	Sessile drop
-SH	21.51	0.52	19.91	0.48	41.42[7]	Glass/MPTMS	Sessile drop

References

1. Yoneda, H. Effect of Substitution on Dipole Moments of Molecules. *Bull. Chem. Soc. Jpn.* **1958**, *31*, 708–714, doi:10.1246/bcsj.31.708.
2. Mansel Davies *Some Electrical and Optical Aspects of Molecular Behaviour 1st Edition*; Irving, R.R.H.M.N.H., Ed.; 1st January 1965; ISBN 978-1-4831-9614-5.
3. John. R, T. *Error Analysis: The Study of Uncertanties in Physical Measurements*; 2nd ed.; University Science Books: Sausalito, CA, 1997;

4. Xue Li; Olivia Niitsoo; Alexander Couzis Electrostatically Driven Adsorption of Silica Nanoparticles on Functionalized Surfaces. *J. Colloid Interface Sci.394*, 26–35, doi:10.1016/j.jcis.2012.11.042.
5. Natarajan, B.; Li, Y.; Deng, H.; Brinson, L.C.; Schadler, L.S. Effect of Interfacial Energetics on Dispersion and Glass Transition Temperature in Polymer Nanocomposites. *Macromolecules***2013**, *46*, 2833–2841, doi:10.1021/ma302281b.
6. Horng, P.; Brindza, M.R.; Walker, R.A.; Fourkas, J.T. Behavior of Organic Liquids at Bare and Modified Silica Interfaces. *J. Phys. Chem. C***2010**, *114*, 394–402, doi:10.1021/jp908444x.
7. Wang, Y.; Hansen, C.J.; Wu, C.-C.; Robinette, E.J.; Peterson, A.M. Effect of Surface Wettability on the Interfacial Adhesion of a Thermosetting Elastomer on Glass. *RSC Adv.***2021**, *11*, 31142–31151, doi:10.1039/D1RA05916E.
